# Dose-dependent interaction of parasites with tiers of host defense predicts “wormholes” that prolong infection at intermediate inoculum sizes

**DOI:** 10.1371/journal.pcbi.1012652

**Published:** 2024-12-06

**Authors:** Andrea L. Graham, Roland R. Regoes

**Affiliations:** 1 Department of Ecology & Evolutionary Biology, Princeton University, Princeton, New Jersey, United States of America; 2 Institute of Integrative Biology, ETH Zürich, Zurich, Switzerland; 3 Santa Fe Institute, Santa Fe, New Mexico, United States of America; Wageningen UR: Wageningen University & Research, NETHERLANDS, KINGDOM OF THE

## Abstract

Immune responses are induced by parasite exposure and can in turn reduce parasite burden. Despite such apparently simple rules of engagement, key drivers of within-host dynamics, including dose-dependence of defense and infection duration, have proven difficult to predict. Here, we model how varied inoculating doses interact with multi-tiered host defenses at a site of inoculation, by confronting barrier, innate, and adaptive tiers with replicating and non-replicating parasites across multiple orders of magnitude of dose. We find that, in general, intermediate parasite doses generate infections of longest duration because they are sufficient in number to breach barrier defenses, but insufficient to strongly induce subsequent tiers of defense. These doses reveal “wormholes” in defense from which parasites might profit: Deviation from the hypothesis of independent action, which postulates that each parasite has an independent probability of establishing infection, may therefore be widespread. Interestingly, our model predicts local maxima of duration at two doses–one for each tier transition. While some empirical evidence is consistent with nonlinear dose-dependencies, testing the predicted dynamics will require finer-scale dose variation than experiments usually incorporate. Our results help explain varied infection establishment and duration among differentially-exposed hosts and elucidate evolutionary pressures that shape both virulence and defense.

## Author summary

Organisms vary in their exposure to parasites and pathogens, such that some individuals breathe in, ingest, or are inoculated (e.g., by insect vectors) with just a few parasites, while others are confronted with large doses. This variation in inoculating dose is likely to affect the odds of successful defense and the duration of infection. For example, individuals confronted with very few parasites might be completely defended, such that infection never establishes. Intuitive though such predictions are, the dose-dependence of the establishment and duration of infection is poorly understood. Here, we use a mathematical model of the three known tiers of immune defenses–i.e., barrier, innate, and adaptive tiers–to investigate how varied inoculating doses interact with immune systems. We find that although the lowest doses are indeed readily controlled, the highest doses induce potent immune responses that curtail infection. As a result, intermediate doses are the ones that can slip past barrier defense yet under the radar of induced defenses. Intermediate doses therefore generate infections of greatest duration. These findings help explain why hosts vary so much in the course of infection and suggest new research directions in epidemiology and immunology.

## Introduction

Following exposure to parasites (here defined broadly to include viruses, bacteria, fungi, protozoa, helminths, or ectoparasites), the dynamics of parasite growth and immune response induction are important determinants of infection success (i.e., the probability of establishing infection) as well as severity and chronicity of infection. For example, hosts that exhibit extremely rapid induction and decay of immune responses are expected to clear infection quickly while minimizing costs of defense [1]. However, the dose of parasites to which a host is exposed is likely to alter these within-host dynamics, both qualitatively and quantitatively: inoculating dose may determine whether infection is established at all [2–6] as well as the duration or severity of infections that do establish [7–10]. Quantitative analysis of dose-dependence in the establishment or mortality risk posed by a given infectious agent is therefore considered a crucial component of public health risk analysis [11]. General principles governing dose-dependence of infection and immunity merit formal investigation.

A prevalent and useful conceptualisation of the quantitative process of infection is the hypothesis of independent action. According to this hypothesis, each individual parasite has the same small chance to initiate an infection, and this chance is independent of how many other parasites are present in the inoculum [12, 13]. Because each parasite can initiate an infection independently if the hypothesis of independent action holds, the cumulative infection probability is predicted to increase monotonically with the inoculum dose [13]. Many systems have been found to conform to the hypothesis of independent action, including classic work on *Haemophilus influenzae* in rats [14] (but see [15]), as well as diverse virus infections in plants [4], insects [3], and fish [5]).

Deviations from the independent action hypothesis have also been conceived and documented. Halvorson [12], who first formulated the hypothesis of independent action, contrasted it with a hypothesis that a critical number of parasites is required for infection. Requiring a critical, or threshold, number is not consistent with parasite individuals acting independently. Rather it constitutes a form of synergy–e.g., when a quorum of bacteria in insects [16] or a quorum of bacteria [17] or protozoa [18] in mammals must cooperatively signal or differentiate to achieve or sustain infection. Mechanistically, such cooperativity could arise directly by signaling among individual parasites, or indirectly [15], for example as the result of the interaction between the parasite population and the immune system of the host, which is focal to this study.

Another apparent deviation from independent action arises when hosts differ in susceptibility (e.g., due to variation among individuals in rates of immune response induction). In this case, the increase in infection success with inoculum dose is flatter than predicted by the hypothesis of independent action, and this slope can be used to quantify the variation in susceptibility among hosts in the population [19–24]. This apparent deviation does not require a lack of independence of parasites during infection, but arises from the population-level effects of susceptibility differences.

Such explanations for the shape of the relationship between inoculum dose and infection success, though informative, do not fully account for the essence of the host-parasite interaction: e.g., the reciprocal quantitative dependence of immune response induction on parasite abundance and of parasite abundance on immune responses. Indeed, even though non-independent action has been invoked as a contrast to independent action, the interactive, mechanistic basis of dose-dependence has rarely been clarified [25]. A notable exception was provided by Pujol et al. [2], in which cooperativity arose as a consequence of the interaction between the parasite population and a single-tiered immune response: in their model, the parasite elicited an immune effector that in turn curbed its growth. Allowing such feedbacks generated complex relationships between inoculum dose and infection success that were consistent with data on poliovirus, among other infections [2]. The interval of time over which parasites were inoculated was crucial to their results, which is perhaps unsurprising, given the time-dependence of immune processes as well as parasite replication.

We were therefore motivated to further explore within-host feedbacks and differential time signatures of different tiers of immune defense. More broadly, we were intrigued by the idea that, due to these feedbacks, infection outcomes could vary, and in the most extreme cases, be bimodal, leading to either immediate parasite clearance or persistent infection. Such bimodal infection outcomes could arise as a consequence of the interplay between stochasticity and within-host feedbacks. Alternatively, they could arise even without stochasticity in a manner similar to Allee effects, which are commonly observed in population ecology and conservation biology [26]. Allee effects arise when positive feedback loops generate ever-higher per-capita growth rates as population density escalates, leading to persistence thresholds for the population (e.g., [27]): below the density threshold, the population will go extinct; above it, the population will persist. Such rules of population ecology could apply to parasite populations growing (or going extinct) within hosts, just as for frog or orchid populations growing (or going extinct) within a forest.

Here, we formulate mathematical models to investigate how the inoculating dose of parasites interacts with multi-tiered immune defenses to determine duration of infection, including whether or not infection establishes at all (i.e., if infection fails to establish, the duration is arguably zero). Like Pujol et al. [2] and a few others (e.g., [28–30]), we thus go beyond the simplification of the classic independent action hypothesis, and mathematically capture more realistic interplay between the parasites and the immune system within the host. The range of inoculating doses that we consider spans many orders of magnitude, because the range of infective doses empirically observed–for example, across various bacterial infections of human hosts–is vast (>6 orders of magnitude) [31], and because we aim to uncover general rules of within-host engagement between any type of parasite and multiple immune components, rather than using a model to explain dose dependence in any one particular infectious disease across a narrower range. We consider that not only do parasites induce immune responses (roughly according to “mass action” of the rates of encounter between parasites and immune cells [32]), but also that different immune system components differ in how and when they are triggered, when they act, and how effective they are (following, e.g., [33, 34]).

Specifically, we model host defenses that are three-tiered, with barrier, innate, and adaptive defenses, as described in mammals (e.g., [35, 36]), but with likely analogues, if not homologues, among other animals as well as plants. These three tiers have distinct temporal properties. First, barrier defenses are constitutive (like skin) or continuously maintained at steady-state levels (like mucosal IgA antibodies exhibiting cross-(microbial)-species reactivity [37]). Such defenses are immediately ready upon exposure but can be overcome or eroded at sufficiently high inoculating doses [38]. Next, non-specific innate defenses such as macrophages consuming microbes are rapidly induced (within hours) and subject to handling time saturation (such that large numbers of parasites can overwhelm them [39, 40]). Finally, specific adaptive defenses like killer T cell or antibody responses are induced more slowly (days to weeks [36]) but are capable of achieving extremely high concentrations that might conquer large numbers of parasites. While these defenses can also have distinct spatial aspects–e.g., local expression of barriers and innate defenses versus more systemic distribution of adaptive defenses–we, for the most part, ignore the spatial aspects in this initial model (aside from in our robustness analysis reported in S1 Code).

Our model of how inoculating dose interacts with these three tiers of defense predicts relationships for the establishment and chronicity of infection with inoculum dose that are inconsistent with the hypothesis of independent action. The inconsistencies we report are more dramatic than in previous studies: not only do we predict that infection success and duration do not increase linearly with dose, we also find profiles with multiple peaks at intermediate parasite doses. The prediction of multiple peaks in the dose-dependence of infection success and duration, if empirically confirmed, is likely to be exacerbated by spatial compartmentalization of induced immune responses, especially for localized infections. If infection duration doesn’t generally increase monotonically with inoculum dose, this would have important implications for the estimation of host susceptibility, for epidemiological dynamics, and for the evolution of strategies for both attack and defense, as we outline in the Discussion.

## Methods

### Model definition

We developed a simple model that describes the growth of the parasite population within the host, and its inhibition by the immune system. Here, we assume no anatomical structure within the host, so, after inoculation, the population dynamics unfold in a single, well-mixed compartment. Because induced innate and adaptive immune effectors migrate to the site of infection, we need not invoke anatomical complexity to study how parasites interact with these three tiers of defense.

Let, *P* denote the number of parasites in this compartment. Parasite population dynamics are described by the following differential equation:

$$dP/dt=\eta (t)+rP-(\gamma_{A}E_{A}+\gamma_{B}E_{B}+\gamma_{C}E_{C})P$$ (1)

Hereby *η*(*t*) is a time-dependent function that captures the exposure of the host. The integral over this function, $\int_{0}^{t}\eta (t\prime )dt\prime$, is the total number of parasites that enter the host, either through natural exposure or experimental inoculation. Because, in this study, we are focusing on the implications of dosing on the within host dynamics, this function is central.

The form of the function *η*(*t*) allows very flexible dosing schedules. Inoculations with a single dose (“bolus”) can be described by function with a high short peak for a short duration, where the height multiplied by the duration gives the overall dose. Alternatively, continuous (“trickle”) or repeated inoculations can also be described.

Once the host has been seeded with parasites, they start to replicate exponentially at a rate *rP* and are inhibited by three immune effectors, the number of which we denote by *E*_*A*_, *E*_*B*_, and *E*_*C*_ and the efficacy of which we denote by γ_A_, γ_B_, and γ_C_. These three immune effector tiers, rather than describing specific actors that can differ among infections or host types, cover the relevant dynamical range of immune responses. In particular, we consider a constitutively expressed barrier effector *E*_*A*_, and two inducible effectors, *E*_*B*_ and *E*_*C*_, that differ in the speed at which they are induced. The slower one, *E*_*C*_, is ultimately more effective (i.e., gamma C is larger than gamma B).

The dynamics of these immune effectors is governed by the following equations:

$$dE_{A}/dt=\sigma_{A}(1-E_{A}/E_{A}(0))-\gamma_{A}PE_{A}$$ (2)

$$dE_{B}/dt=\frac{\sigma_{B}(1-E_{B}/K_{E_{B}})E_{B}P}{h_{B}+P}$$ (3)

$$dE_{C}/dt=\frac{\sigma_{C}(1-E_{C}/K_{E_{C}})E_{C}P}{h_{C}+P}$$ (4)

*E*_*A*_ is constitutively expressed at the level *E*_*A*_(0). This effector is depleted when it kills the parasite at a rate *γ*_*A*_*PE*_*A*_. It is replenished at a maximum rate *σ*_*A*_. The other two effectors. *E*_*B*_ and *E*_*C*_, are induced at rates that depend on the parasite load *P*. At low parasite loads, *P* and low levels of *E*_*B*_ and *E*_*C*_, they are induced at exponential rates *σ*_*B*_*P* and *σ*_*C*_*P*, respectively. The parameters *h*_*B*_ or *h*_*C*_ denote the parasite loads at which these two rates are half the maximum. The induction rate goes to zero if the levels of the immune effectors reach their respective carrying capacities $K_{E_{B}}$ and $K_{E_{C}}$. Unlike *E*_*A*_, *E*_*B*_ and *E*_*C*_ are not assumed to be decimated by killing parasites. The dynamics of *E*_*B*_ and *E*_*C*_ are structurally identical, but they differ in terms of the speed of induction (*σ*_*B*_ and *σ*_*C*_) and maximum efficacy ($\gamma_{B}K_{E_{B}}$ and $\gamma_{C}K_{E_{C}}$)

### Model parameterization and scenarios

The model is parameterized generically. This means that, rather than simulating any specific infection, we choose our parameters such that they recapitulate the typical time scales of the generation of the three tiers of immune responses upon exposure.

In the first instance, we assumed that, following arrival at an array of inoculating doses, the parasite replicates at a per capita rate of 1 per day, which corresponds to a doubling time of approximately 0.7 days, or a 10-fold increase in approximately 2.3 days. Parasite growth is not assumed to be limited by a carrying capacity. When we instead simulate macroparasitic infection, we assume that parasites enter the hosts at various numbers, but do not replicate. We also ran simulations for singular ("bolus") or continuous ("trickle") exposure to both micro- and macroparasites.

The first, barrier tier of the immune system is constitutively expressed at the arbitrarily chosen level of 100 effectors, *E*_*A*_. As for mucosal IgA antibodies, by exerting their effect, they are depleted, and at a rate that depends on the inoculum size, leading to either an immediate depletion of the effectors when facing a large inoculum dose, or to a fast clearance of a small inoculum. Once the parasites are cleared, these first-tier effectors grow back to the initial level of 100 at an initial rate of *σ*_*A*_ = 30 per day. The rate is reduced linearly until the homeostatic level is reached.

The second tier of the immune system, *E*_*B*_, is conceived as innate immune components that are initially set to a single effector. They are stimulated by parasites that overcome the first-tier response to proliferate/recruit at a logistic rate that depends on the parasite load. A single second-tier effector is assumed to be slightly less effective than a single first-tier effector. These second tier effectors can reach a carrying capacity of 10’000. At this level they are still assumed to be 20% less potent than the barrier response at its constitutively expressed level, to capture handling time constraints for effectors such as macrophages [39, 40] compared to mucosal antibodies at the barrier [41, 42]. Unlike the first-tier effectors, however, second-tier effectors are not depleted by exerting their effect. Thus, the second tier is effective across a larger range of parasite doses.

The third tier of the immune system, *E*_*C*_, is conceived as adaptive immune effectors that are assumed to differ from the second tier only quantitatively, in terms of both a slower induction rate and higher effector efficacy. They are elicited to proliferate by the parasites that overcome the first-tier responses at a rate that is two orders of magnitude lower than the induction rate of the second tier. Their per-effector potency to clear parasites is assumed to be the same as that of the first-tier effectors. Their carrying capacity is set to 10^6^ but in practice they stop proliferating before that cap because they typically lead to fast parasite clearance.

In these model formulations for the innate and adaptive tiers of defense, we assume a dependence of the induced immune responses on the dose of antigen. For both innate and adaptive responses, there is clear evidence for such dose-dependence. For example, the magnitude of CD8+ T cell responses in mice is affected by the dose of the antigen (e.g., see Fig 1 in [43]). The magnitude of antibody responses are also clearly affected by dose. A prominent case in point comes from the phase I/II trials for the mRNA vaccines against COVID that showed a clear dose-dependence of the antibody response (e.g., see [44] and [45]; in this context, we refer to the dose of the vaccine antigen rather than of the whole parasite, but those are immunologically similar in terms of quantitative effects of increasing antigen exposure on the induced immune response). The magnitude of some innate responses also depends on the antigen dose [46], as reflected in the implementations of innate immunity in previous studies [30, 47] and in our model.

Such evidence is the basis for our assumption that parasite load affects the quantity of induced immune effectors. Interestingly, it has been established that the dynamics of the induced response for some adaptive immune components can unfold according to an intrinsic, parasite-load-independent program after initial activation [48]. Our implementation of the adaptive response does not take this program into account, but dose-dependence of the different stages of induced lymphocyte responses (from activation to proliferation) would be an interesting avenue to explore in future work.

We deviate from our default parameterization to investigate the infection dynamics in immune-tier “knockout" hosts. These tier "knockouts" are implemented by setting the initial concentration of the respective immune effectors, *E*_*A*_, *E*_*B*_, or *E*_*C*_, to zero. This procedure mirrors knocking out genes responsible for various immune components in mice in experimental immunology [49], but is, due to our conceptual approach, more generic and cleaner because it is without pleiotropic effects on other traits.

### Model implementation

We implemented the deterministic population dynamical model in Eqs 1–4 in the R language for statistical computing [50] using the function lsoda in the package deSolve [51].

We also implemented a stochastic version of the model using the implementation of the Gillespie algorithm in the R-package adaptivetau [52] that implements the adaptive tau-leaping approximation for simulating the trajectory of a continuous-time Markov [53]. For the stochastic implementation, the logistic growth terms in Eqs 3 and 4 have been partitioned into two terms, on describing the population expansion ($\frac{\sigma_{B}E_{B}P}{h_{B}+P}$ and $\frac{\sigma_{C}E_{C}P}{h_{C}+P}$), the other describing death due to crowding ($\frac{\sigma_{B}(-E_{B}/K_{E_{B}})E_{B}P}{h_{B}+P}$ and $\frac{\sigma_{C}(-E_{C}/K_{E_{C}})E_{C}P}{h_{C}+P}$). (This partitioning of the growth terms is generally required for the stochastic implementation of logistic growth to prevent the population turnover to be zero at the carrying capacity.)

For all details on our simulation models, the parameterization, and the alternative, more complex model we used to assess the robustness of our inferences, please refer to code and results that are provided in S1 Code.

## Results

**Fig 1:**
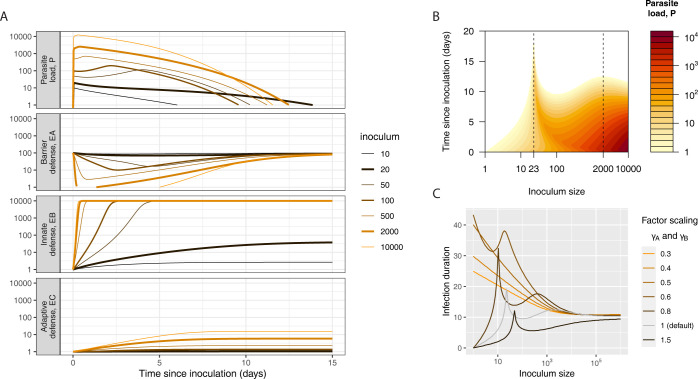
**(A)** Time courses of parasite load (top row) and immune effectors of all three tiers (second, third, and fourth rows, corresponding to barrier, innate, and adaptive tiers, respectively) for varying inoculum sizes (doses). Intermediate inoculum sizes cause the longest infections due to how they interact with the series of defense tiers. The parasite loads in the top row start out at 0 and ramp up quickly to the inoculum dose within one hour, consistent with our implementation of the bolus inoculation (see S1 Code). **(B)** Time-course of parasite load for the varying doses displayed as a contour plot. (These are the same simulation data as shown in (A)). In this contour plot, it is more clearly visible that infection duration (in days since inoculation, on the y-axis) is longest at doses 20 and 2000, even if peak parasite load (colored according to heatmap P, at right) is highest in hosts receiving the largest dose. These simulations are based on the default parameterization of our model (see Model Parameterization and Scenarios as well as S1 Code.) **(C)** Infection duration versus inoculating dose for varying strengths of the first- and second-tier immune responses. The efficacy parameters of the first and second tier defenses, *γ*_*A*_/*γ*_*B*_, have been scaled to keep their relative efficacies constant. The two peaks of infection duration are at lower doses for weaker immune responses, and for very weak responses they disappear completely, showing that the peaks in the relationship between duration and inoculum size are determined by the strength of first- and second-tier defenses.

We studied the dose-dependence of immune response induction and within-host dynamics of infection using a mathematical model. The model describes how the parasite invades the host, replicates in it, and is confronted with three tiers of defense–roughly corresponding to the constitutively maintained barrier, the induced innate, and the induced adaptive tiers. Initially, we ran deterministic model simulations varying the inoculum size (dose) across four orders of magnitude. Parasites and the three immune components exhibited diverse dose-dependent dynamics (**Fig 1**), as follows.

The dynamics of parasites (**Fig 1A**) were non-monotonic: very low doses generated low peak parasite densities and short-lived infections; at higher doses, parasites persisted longer, with a peak in duration at the inoculum dose 23; at yet higher doses, infection duration decreased approximately two-fold, only to increase and peak again at an inoculum dose of 2000; beyond that, infection duration decreased again (**Fig 1B**). We also found that the multi-peaked relationship between inoculating dose and duration was robust to variation in the efficacy of barrier and induced defenses, with more potent defenses simply reducing the height of the peaks (**Fig 1C**).

We also tracked immune effector dynamics separately for the three tiers of defense. The effectors of the barrier defense (*E*_*A*_ in **Fig 1A**) exhibited a depletion that depended on the size of the inoculum. For inoculum sizes of 2000 and larger, this depletion was complete. By contrast, dynamics of the rapidly inducible (innate-like) and slowly-inducible (adaptive-like) defenses varied in a more straight-forward fashion with dose. Innate immune effectors (*E*_*B*_ in **Fig 1A**) were induced increasingly rapidly with increasing dose, up to a maximum level of defense. Intermediate doses thereby induced middling innate defenses. Adaptive immune effectors (*E*_*C*_ in **Fig 1A**) likewise exhibited straightforward dose dependence: both rates of induction and peaks of adaptive immune effectors increased monotonically with increasing parasite dose.

Together, these results suggest that intermediate doses achieved long duration of infection by escaping the first or second tier defenses and then inducing little further defense. In this way, such doses found a “wormhole” through 3-tiered host defense systems. (Hereby, we are referring to a as described in the movie “Stargate,”as a hypothesized portal through the space/time continuum, rather than a hole in old cupboards.)

**Fig 2:**
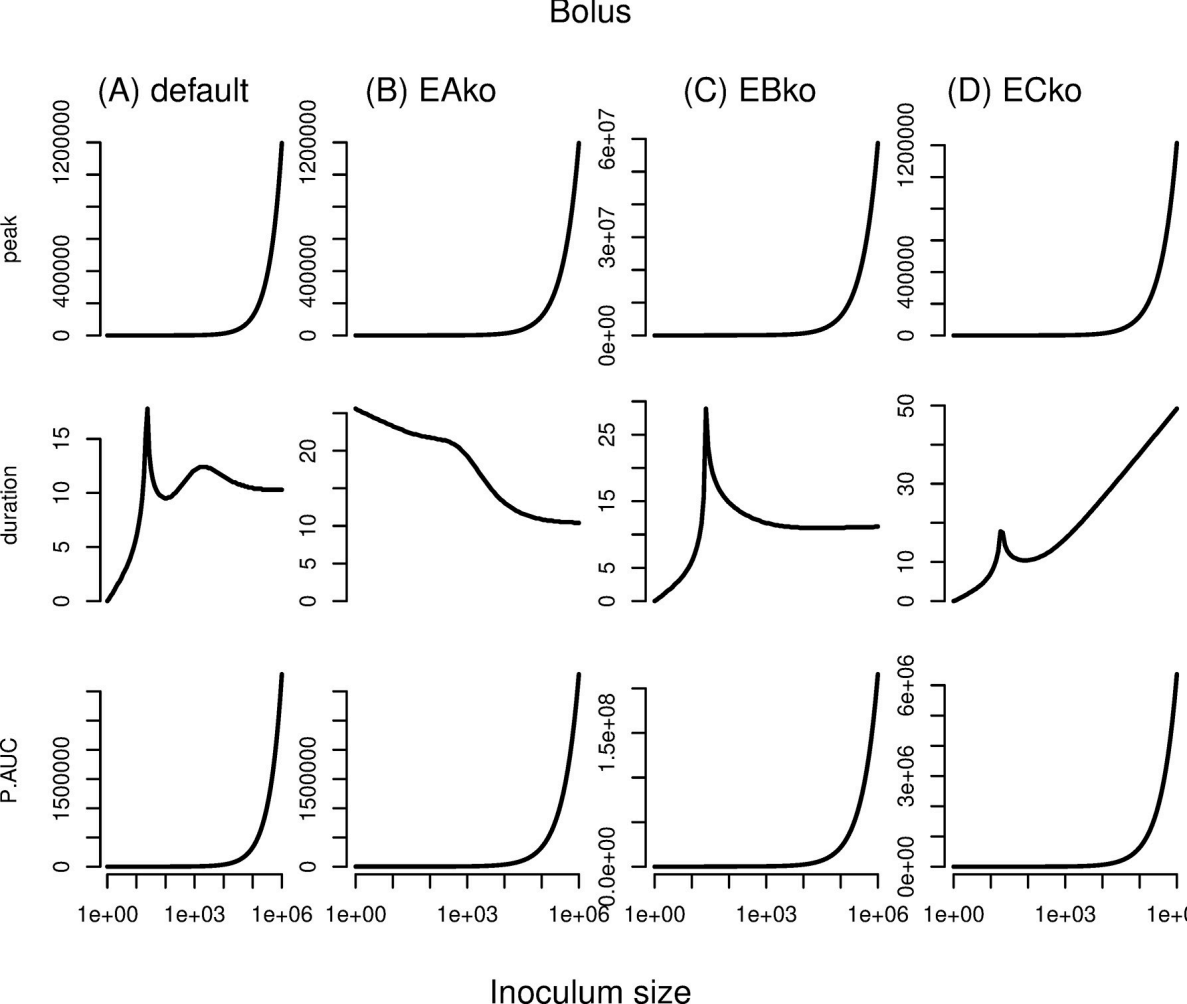
Peak parasite load (top row), infection duration (middle row), and cumulative parasite load (AUC, or area under the curve; bottom row) statistics for deterministic simulations across a range of inoculum sizes in 4 host types: (A) wildtype (“default”), (B) barrier knockouts (“EAko”), (C) second-tier (innate) knockouts (“EBko”), and (D) third-tier (adaptive) knockouts (“ECko”). Note the non-monotonic profiles of infection duration, especially in wildtype or innate-knockout hosts ("EBko”).

We next systematically studied the peak parasite load and infection duration within 4 host types: “wild type” hosts with all 3 lines of defense intact; “barrier knockout” hosts lacking the first tier defense; “innate knockout” hosts lacking the rapidly inducible tier of defense; and “adaptive knockout” hosts lacking the slowly inducible tier of defense (**Fig 2**). To obtain sufficient resolution, we studied dose variation across six orders of magnitudes of inoculum size in each of these hosts.

In wild-type “default” hosts (**Fig 2A**), we found that peak parasite load (top row) exhibited straightforward dose-dependence, increasing gradually with increasing dose. The duration of infection (second row), in contrast, exhibited non-monotonic dose dependence. Most interestingly, infection duration peaked twice across dose, as described above. The cumulative parasite load (third row) shows the same pattern as the peak parasite load. Thus, the non-monotonic pattern in duration is over-ridden by increases in peak parasite load to generate increases in cumulative parasite load with increasing dose.

The three knockout host types provide insight into the causes of these patterns. Generally, for all types of knockouts (**Fig 2B–2D**), the average duration of infections becomes longer across the range of doses compared to wild-type (see y-axis ranges).

Barrier knockout hosts exhibited simpler dose dependence than the wild-types, with peak parasite load (top row) increasing with increasing dose (**Fig 2B**). Most notably, however, infection duration (second row) did not display two peaks anymore but continuously decreased with dose. This decrease became more pronounced beyond a inoculation dose of approximately 500, echoing the second peak in the duration profile in the wild-type hosts (**Fig 2A**), while the first peak had disappeared. This change of pattern can be explained by the fact that knocking out barrier defenses deletes the transition between first and second tier that is responsible for the first peak of the duration at low inoculum doses.

Knocking out innate responses (second tier), in contrast, makes the second peak in infection duration (second row) at high inoculum doses disappear (**Fig 2C**). The profiles of peak parasite load (top row) did not increase gradually anymore, but displayed jumps at an inoculum dose of 20, at which barrier responses are overcome and adaptive immune responses are triggered.

The patterns in adaptive immune knockouts are similar to those in innate immune knockouts: the second peak in the profile of infection duration versus dose (second row) disappears (**Fig 2D**). Unlike in innate immune knockouts, however, the infection duration does not continuously decrease with further dose escalation, but instead turns around and increases again. This difference in the patterns between innate versus adaptive immune knockouts may be due to the lower potency of innate compared to adaptive immune effectors in our model.

These results show the link between the peaks in the profile of infection duration and tier transitions, and support a central role for tier transitions in explaining why intermediate doses of parasites lead to infections that persist longest. In all cases, cumulative parasite load exhibited the same monotonic dose-dependence as peak parasite load.

**Fig 3:**
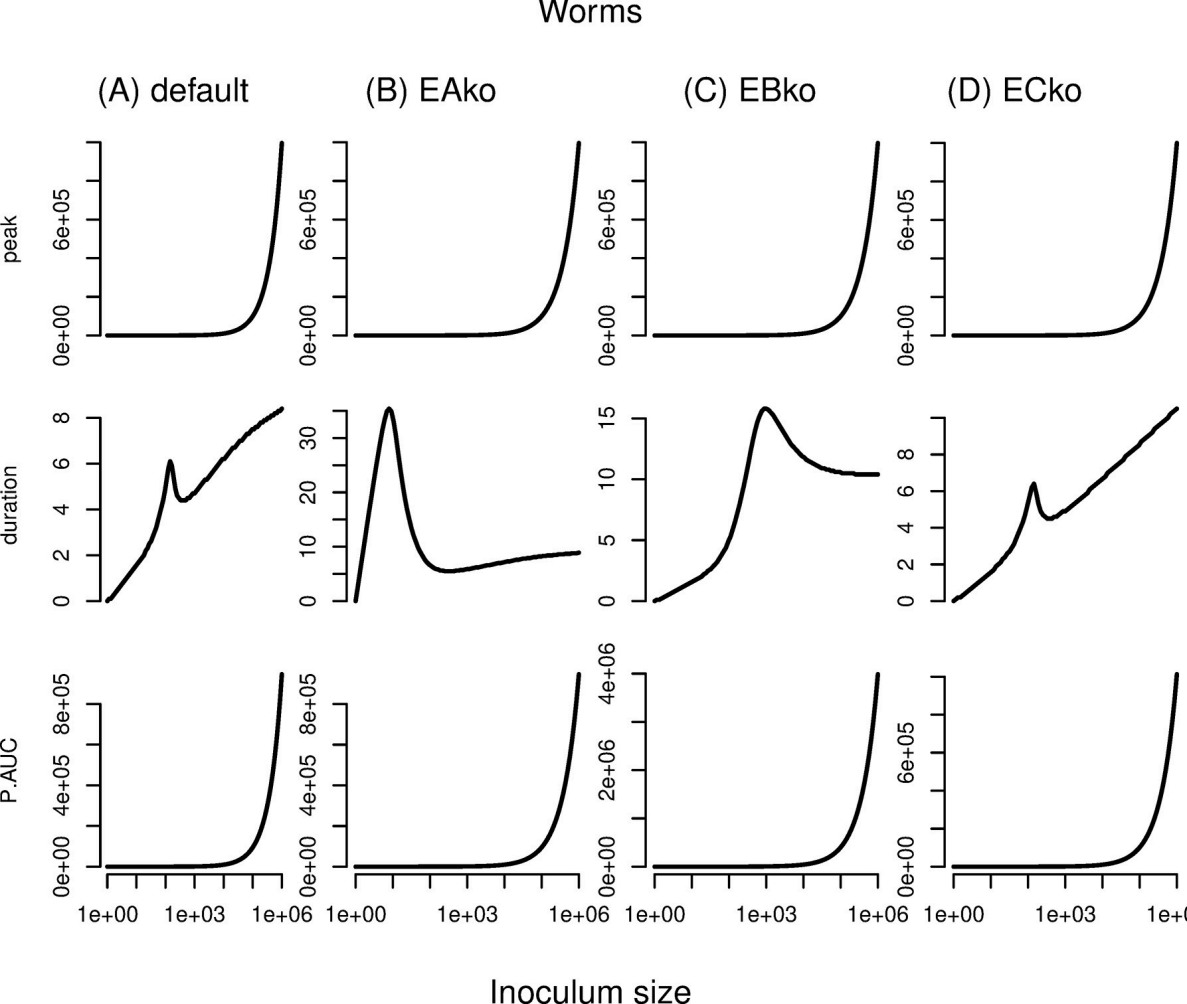
Peak parasite load (top row), infection duration (middle row), and cumulative parasite load (AUC, or area under the curve; bottom row) statistics for deterministic simulations across a range of inoculum sizes in 4 host types: (A) wildtype (“default”), (B) barrier knockouts (“EAko”), (C) second-tier knockouts (“EBko”), and (D) third-tier knockouts (“ECko”). Here, all parasites are non-replicating macroparasites, shorthanded here as worms, that arrive all at once in a bolus inoculation.

Our final set of analyses of the deterministic system of equations aimed to assess the generality of the dose-dependent dynamics (**Fig 1**) and summary statistics (**Fig 2**) described thus far. We compared microparasites versus macroparasites (which, by definition, are non-replicating within a given host), and different patterns of exposure (a bit each day in a “trickle” versus all at once in a “bolus”). For the macroparasite bolus exposures, the qualitative conclusion that duration depends non-monotonically on dose was the same as for microparasites, but details differed: e.g., intermediate doses generated a local, if not global, maximum for duration of infection even in barrier and innate response knockouts (**Fig 3**); the macroparasite dose-dependence is remarkably parallel to the microparasite dose-dependence in this system. Trickle exposures induced complexities, especially when parasites continued to arrive after considerable immune response induction and in hosts with a tier of defense knocked out (**S1 and S2 Figs**). In no case was duration of infection monotonically associated with dose.

**Fig 4:**
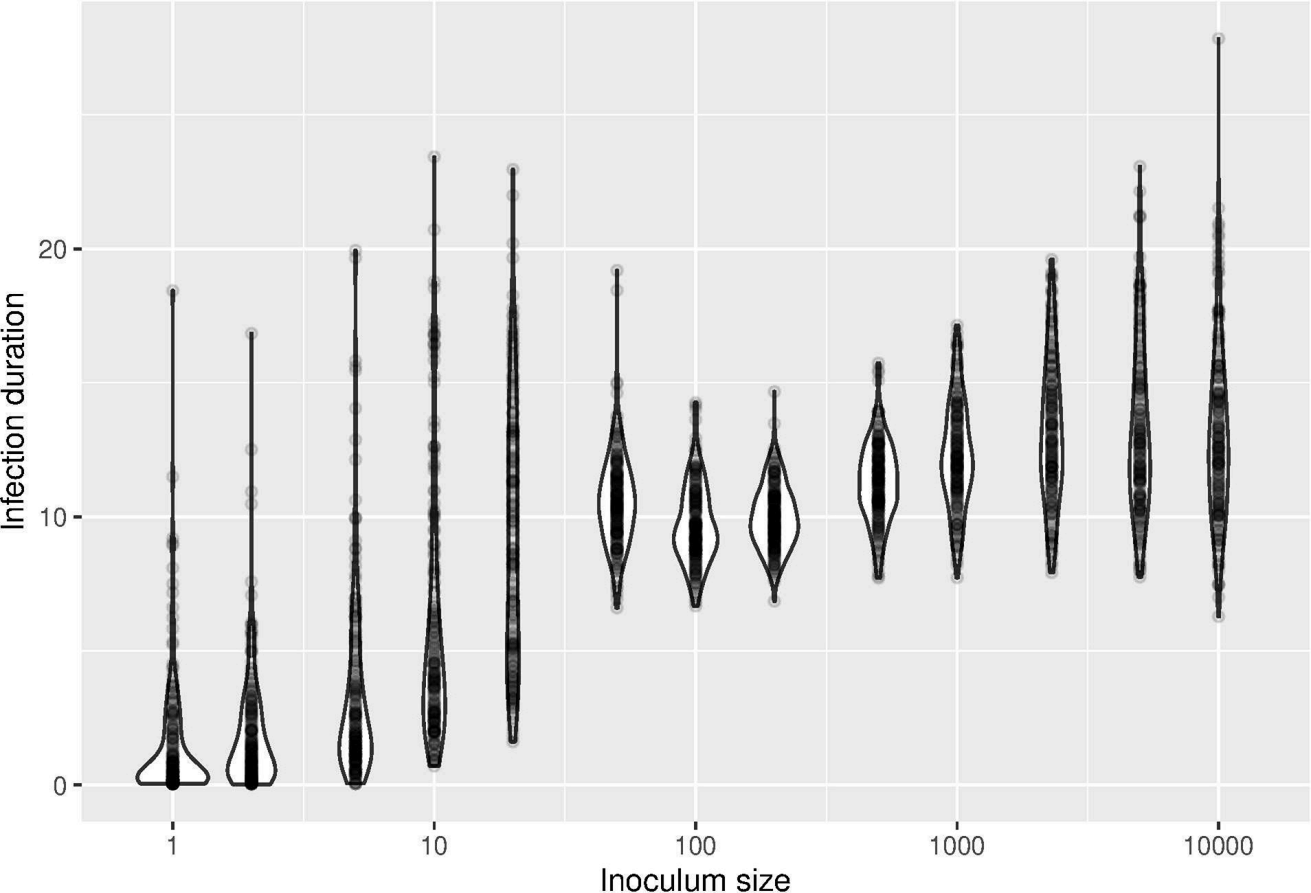
Infection duration versus inoculum size in stochastic simulations of the model, again showing non-monotonic changes in infection duration with increasing dose. Note that inoculum sizes of ~50 that overcome the first-tier responses lead to less variable durations than inoculum sizes of 20.

We next undertook stochastic simulations, to investigate how random variation in the processes hypothesized in our model would impact outcomes. We particularly focused on the response variable of infection duration, and we used finer-grain variation in inoculum size than before, but again across four orders of magnitude of dose. Consistent with the deterministic results, the stochastic results show non-monotonic dose dependence of infection duration with two peaks and tremendous variance associated with overcoming the first two tiers of defense. Again, doses intermediate between the extremes often generated the most persistent infections (**Fig 4**).

**Fig 5:**
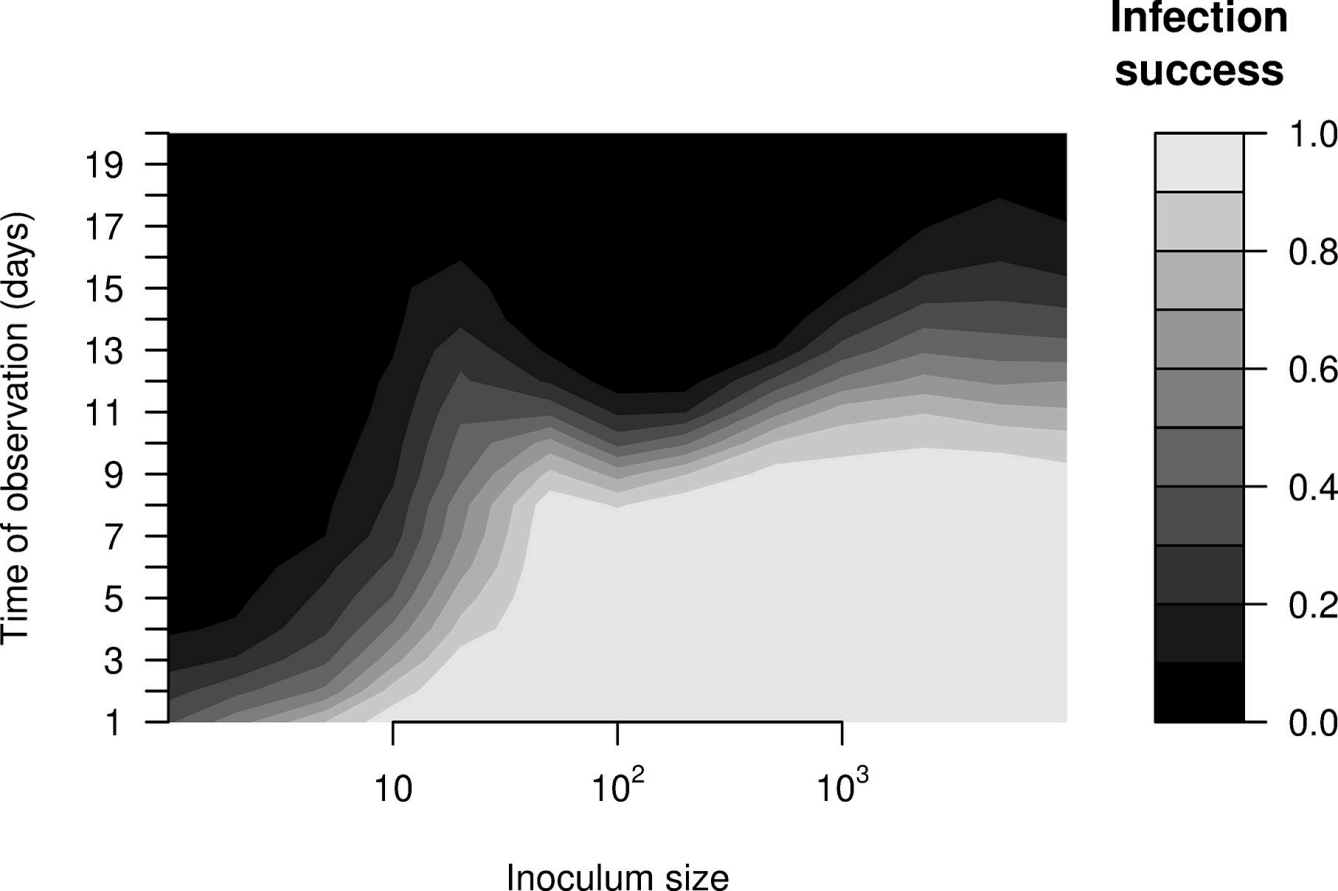
Infection success against inoculum size and time of observation. As expected, infection success increases overall with the inoculum size, and is higher when observed sooner. Note, however, the non-monotonic, double-peaked profile of infection success versus inoculum for observation times of 10–15 days.

Finally, we drew upon our stochastic formulation to investigate how infection success depends upon inoculum size. We yet again observed non-monotonic, two-peaked dose-dependence, but time of observation emerged as crucial to the inference, as follows. Observed early after exposure, infection success appears to have a sigmoidal relationship to dose, with increasing success with dose up until a first peak. Observed later after exposure, hosts receiving high doses and thus more stimulation of inducible defense began to clear their infections, which generated a double-peaked relationship between dose and infection success (**Fig 5**). In other words, even infection success has two peaks at different intermediate doses, but detecting that relationship depends upon the timepoint at which hosts are observed and thus declared infected or not.

Further simulations revealed that the stochastic version of our mathematical model can display bimodal variation in infection dynamics, such as those observed previously [6, 33]. **S3 Fig** shows simulations of the time course of the parasite population in hosts, in which the second-tier innate responses are assumed to be knocked out. At the inoculum size that breaches the barrier response, the time courses are extremely variable, leading to bi-modal infection duration distributions. Finally, stochastic simulations of the model also revealed that, in wild-type (i.e., immunologically intact) hosts, both peak and cumulative parasite load escalate monotonically with increasing inoculum size (**S4** and **S5 Figs).**

In a final suite of both deterministic and stochastic models, we investigated the robustness of our findings to the particular implementation of the three immune response tiers in our model. To this end, we developed an alternative model (see S1 Code). In the alternative model, we added an exterior compartment to allow the barrier defense to act in its own spatial compartment, we assumed alternative stimulation terms of the innate defense (drawing upon the approach of [47] and [30]), and we linked the stimulation of the adaptive defense to the innate defense, leading to a clearer separation in the timing of these two immune response tiers. In each case, the qualitative patterns we observed using the original, simpler model above held: dose-dependence in duration and infection success were in all cases non-monotonic (e.g., see **S6** and **S7 Figs**).

## Discussion

Immunoepidemiology links the dynamics of parasites within the host to their epidemiological spread. The two most essential parameters at this interface between immunology and epidemiology are exposure and susceptibility of hosts. Complicating immunoepidemiological analysis, however, is the fact that exposure and susceptibility are not independent of each other. Indeed, the number of parasites (or parasites per unit time [2]) to which hosts are exposed (here, inoculating dose) may in fact determine how susceptible the host is to infection *per se* [19–24], as well as susceptibility to chronic infection or severe symptoms [7, 8, 54–56]. Studies of dose-dependence are therefore of applied relevance to risk analysis [11]. In our view, dose-dependence studies are also at the very heart of immunoepidemiology, because they enable testing of basic science hypotheses such as the independent action of microbes [12, 13] or the optimal blend of constitutive and inducible defenses [57]. The value of studies of dose is greatly enhanced when physiological mechanisms are incorporated [25], as we have attempted here, with coarse but realistic incorporation of three tiers of host defense meeting varied inoculating doses.

Using a deterministic model that considers immune responses dynamically, we found that both the establishment and duration of infection exhibited complex dose-dependence. Two intermediate inoculating doses generated infections of longest duration, while very low and very high doses led to infections of shorter duration. We found that this is explained by certain doses being sufficient to clear the first and even the second tier defenses but then only slightly/slowly inducing subsequent tiers of effectors. This pattern is supported in at least some empirical systems (e.g., dose-dependent induction and efficacy of immune defenses by malaria parasites [40, 58]). Similarly, vaccine-induced immune responses are maximal at intermediate doses of the attenuated parasite strains included in vaccines [30].

Our findings thus suggest that parasites arriving at intermediate doses may be more likely to establish and persist in a host. Simulated knockout of barrier or innate defenses turned the complex dose dependence with two peaks into simpler form, usually with a single peak of chronic infection associated with intermediate doses. Our stochastic simulations revealed threshold doses of approximately 23 and 2000 (for the parameters used) around which duration peaked in many cases. This corresponds to the dose necessary to clear either barrier or innate defenses, and the variation suggests within-host feedbacks and stochasticity leading to variation in trajectories of infection.

We note that the hypothesis of independent action [12] generates a very particular, monotonically-increasing profile of infection success versus inoculum dose. Such monotonic escalation of infection success or mortality rate with inoculating dose is empirically supported in various host-parasite systems (e.g., [3–5]) and provides important public health risk assessment tools [11], as cataloged on the Quantitative Microbial Risk Assessment Wiki (QMRI). Yet there are exceptions: for example, mortality risks associated with *Naegleria fowleri* and nontyphoid *Salmonella typhimurium* infections escalate more rapidly with dose than the considered monotonic models of QMRI can capture. In the case of *S*. *typhimurium*, the dose-response data appear downright non-monotonic. Such exceptions could be due to experimental uncertainty, but may also be taken as motivation to use mathematical tools to explore non-monotonic relationships more systematically, as we have done here.

In any case, the results of our analysis are inconsistent with the generality of the hypothesis of independent action. We go beyond other studies that conceptualize deviations from the independent action hypothesis [2, 15, 29] by revealing rules of within-host ecology that generate non-monotonic profiles of infection success and duration with escalating dose. Furthermore, our model suggests that the “one is enough” corollary of the independent action hypothesis, in which a single parasite has a considerable chance of establishing infection [59], is not general and may depend upon the absence of key defenses. Importantly, our model assumed no cooperativity to the parasites themselves. Instead, an “apparent cooperativity" arose due to dynamical interactions of the parasite population with each of the tiers of host defense.

Indeed, our central finding is that the first and second tiers of immune defenses are crucial to both the non-independent action of parasites and chronicity-permitting “wormholes” in host defense. It is not new to suggest that constitutive defenses are as important as inducible in explaining biotic interactions [60] including when defenses against infection incur costs (e.g., [57, 61]). What is new here is the inference that this suite of defenses may be what confers complex dose-dependence. Others have emphasized the importance of immune response growth terms that are independent of parasite load [28, 33, 34], and we extend this to barrier defense biology which may, in general, be central to predicting the probability and course of infection when host exposure varies. We find that these conclusions are robust to various alternative model formulations that make more complex and realistic assumptions about the anatomical compartment in which a barrier might act, the functional form of innate response induction, and the dependence of adaptive response induction upon the innate tier (see S1 Code).

Relevant barrier defenses are diverse, ranging from constitutive physical and chemical barriers (such as skin or stomach acid) through to continuously produced mucins, antimicrobial peptides (AMPs), or IgA on mucosal surfaces [36]. Mucus, for instance, provides vital multi-pronged barrier defense across the enormous surface areas of the gut, lung, and reproductive tract [62]. Although it is produced by B cells, IgA is continuously secreted in mass quantities, is often antigen-independent (and thus doesn’t require induction of an adaptive response), cross-reactive across many microbial antigens, and also functions as a barrier defense [37]; such IgA can, for example, alter establishment of gut commensals [41] or prevent infection by pathogenic pneumococci [42]. Dose-response studies of barrier defenses are rare but have revealed, for example, that even barriers of intact skin and conjunctival mucosa are overcome when a host is exposed to sufficiently high doses of *Leptospira* bacteria [38].

There are, of course, important caveats to acknowledge, but some of these also lead to insights and predictions for the evolution of immune systems. For example, we modeled just three coarse tiers, but in reality, within each coarse tier, there are finer grained tiers. Such granularity is evident in differential dependence of different induced defenses upon parasite load (e.g., [33, 34]) or immunisation rate [63] as well as varied innate defenses of, for example, the lung [64, 65] or the diversity of T cell types that do not neatly fall in the classic innate vs adaptive categories [66]. This array of finer tiers, including tiers of extremely rapidly induced defenses, imply that “constitutive defenses” actually are part of a continuum [36] of rates of defensive readiness. Our model revealed that three coarse tiers leave vulnerabilities around the barrier and innate defenses; perhaps more realistic redundancies and finer-scaled tiers would mitigate such vulnerabilities. It is tempting to speculate that evolution has led to such redundancies and fine-scale tiers for the very reason that tier transitions open vulnerabilities. Nonetheless, animals such as cnidarians [67] are thought to have just two coarse tiers of defense (barrier & innate). It would be fascinating to investigate whether there are systematic differences in the relationship between inoculum dose and infection success or chronicity across hosts with two vs. three coarse tiers of defenses. Our study suggests that in organisms with only two tiers of defense, there should be only one “wormhole” through which parasites might travel. It would also be useful to test whether/how multiple anatomical compartments might interact with tiers of defense to affect dose-dependence of infection duration, perhaps by comparing localized versus systemic infections. The fact that plants have barrier defenses as well as synergistic recognition and effector defenses [68] plus adaptive RNAi capability (e.g., [69]) suggests that our three-tier predictions might be interesting to test in plants as well as animals.

Furthermore, dose-dependent probabilities and durations of infection are likely to have implications for the evolution of virulence, so we second Schmid-Hempel and Frank’s suggestion [31] that this is a rich area for further research. Virulence is generally defined as fitness effects of infection upon the host, and is hypothesized to trade off against transmission potential–i.e., parasites that kill hosts too quickly might lose out on transmission opportunities, while parasites that cause little damage to the host might transmit a lot, as long as they avoid rapid immune clearance [70]. It would therefore be interesting to investigate how non-monotonic dose-dependence of the probability and duration of infection (as reported here) might affect host fitness, parasite transmission, and thereby the evolution of virulence. For example, would the highest doses accelerate host death, enhancing the benefit to parasites arriving at an intermediate dose? Such investigation is beyond the scope of this study, however, in part because the effects of parasite load and infection duration on host fitness are complex and system-dependent. For instance, virulence might be maximized by maximal cumulative parasite load in some systems, but other relationships are conceivable: e.g., if a threshold number of parasites triggers death [71] or if virulence is actually maximized at low parasite burden due to severe immunopathology [72].

The implications of dose-dependence for transmission are likewise complex but worthy of future investigation. For instance, we found that duration, but NOT cumulative parasite load, is maximized at intermediate doses. In empirical systems where cumulative parasite load is linearly related to cumulative transmission probability, transmission would therefore not be predicted to be maximized at intermediate doses. We would nonetheless still advocate for the importance of dose-response studies in such systems, to better predict how exposure maps onto onward transmission even in simpler systems. However, a linear relationship between cumulative parasite load and cumulative transmission probability does not always hold.

Indeed, there are examples of parasites for which duration *per se* rather than cumulative parasite burden maximizes transmission. For instance, when the infectious dose is small, as for norovirus, the sheer number of days of shedding exceeds the importance of load in predicting the risk that a host will seed an outbreak [73]. Human Immunodeficiency Virus (HIV) provides another example, because of the nonlinear relationship between set-point viral load and the probability of onward transmission [74] as well as the disconnect between transmission probability and the HIV load in the host at the time of transmission [75]. Furthermore, because we found that infection success is also maximized at intermediate doses, this arguably represents a strong effect of dose upon onward transmission because an effective duration of zero could preclude the development of any differences in cumulative parasite load. Such non-monotonic dose-dependence in infection success, if borne out experimentally, would therefore be likely to affect transmission and virulence of all parasite species.

We look forward to further work on the evolutionary ecology of dose-dependence, both theoretical (to further explore the rules governing the evolution of defenses and virulence) and empirical (to test the predictions). One testable prediction is the two-peaked dose-dependence in infection duration that should hold if there are three tiers of immune responses with two clearcut transitions among them. Empirical dose-response studies with a wide range of doses could reveal such patterns. Empiricists designing dose-response studies are often logistically constrained to select just a few doses, however, and it may be challenging to identify a suitable range that will reveal any hidden nonlinearities, or even non-monotonicities. Indeed, the dose that is intermediate will vary hugely across parasite taxa, in ways that may map on to their life histories, virulence, and/or extent of spatial compartmentalization [31]. The predictions generated here might thus best be tested via experimental approaches that track bottlenecks of founder bacteria in hosts deficient in immune defenses of various speeds of activation and efficacy–e.g., barcoded isogenic *Listeria monocytogenes* combined with host immune manipulation as in the elegant work of Zhang et al. [76]. One could also investigate how dose-dependencies change in hosts with altered immune systems. Actual *in vivo* barrier knockouts analogous to our *in silico* barrier knockouts may be rarely practical, but knockdowns may be: as in the work on leptospirosis of Gostic and colleagues [38], barrier defenses like skin or mucosa could perhaps be experimentally eroded for dose-response studies across a broader array of infections.

We conclude by noting that empirically-grounded theory has made a great deal of recent progress in revealing rules of host-parasite interaction. These include stochasticity around inoculating dose thresholds or other early dynamics that lead to life or death for the host [6, 77]. Another insight that has emerged from recent theory is that acute and chronic infections can emerge from the same set of equations so long as suitable feedback mechanisms (e.g., between parasite growth and induced immune defenses, or among different induced immune responses) are built in [78–80]. Here, we add that complex dose-dependence of the probability and duration of infection also arises from such feedbacks when combined with the stochasticity inherent in the within-host parasite population dynamics. We note that, in the present work, variable or bimodal infection outcomes are not technically related to the Allee effect because our model does not feature multiple equilibria that characterize this effect. Nonetheless, Allee effects and other dynamical patterns arising from within-host feedbacks may provide a general framework for understanding rules governing patterns of dose-dependence across a wide array of infectious diseases (such as those represented in Quantitative Microbial Risk Assessment [10, 11]), including to help explain observed deviations from monotonic dose-dependence. These tractable insights from within-host dynamics are likely to have major implications for the immunoepidemiology of diverse infections as well as the evolution of defense systems. These are exciting times indeed for quantitative studies of host-parasite interactions.

## Supporting information

S1 Fig Peak parasite load (top row), infection duration (middle row), and cumulative parasite load (AUC, or area under the curve; bottom row) statistics for deterministic simulations across a range of inoculum sizes in 4 host types: (A) wildtype (“default”), (B) barrier knockouts (“EAko”), (C) second-tier knockouts (“EBko”), and (D) third-tier knockouts (“ECko”). Here, all doses arrive slowly over time, in a trickle rather than bolus inoculation.
(TIF)

S2 Fig Peak parasite load (top row), infection duration (middle row), and cumulative parasite load (AUC, or area under the curve; bottom row) statistics for deterministic simulations across a range of inoculum sizes in 4 host types: (A) wildtype (“default”), (B) barrier knockouts (“EAko”), (C) second-tier knockouts (“EBko”), and (D) third-tier knockouts (“ECko”). Here, all parasites are non-replicating macroparasites/worms, that arrive slowly in a trickle inoculation.
(TIF)

S3 Fig Stochastic simulations of the time course of the parasite population in hosts in which the second-tier innate responses are assumed to be knocked out. (TIF)

S4 Fig Peak parasite load from stochastic simulations of wild type hosts, in which peak parasite load escalates monotonically with increasing inoculating dose. (TIF)

S5 Fig Cumulative parasite load (AUC, or area under the curve) from stochastic simulations of wild type hosts of wild type hosts, in which cumulative parasite load escalates monotonically with increasing inoculating dose. (TIF)

S6 Fig Peak parasite load (top row), infection duration (middle row), and cumulative parasite load (AUC, or area under the curve; bottom row) statistics for deterministic simulations in our alternative model, across a range of inoculum sizes in 4 host types: (A) wildtype (“default”), (B) barrier knockouts (“EAko”), (C) second-tier knockouts (“EBko”), and (D) third-tier knockouts (“ECko”). Here, all parasites are microparasites that arrive all at once in a bolus.
(TIF)

S7 Fig Infection success against inoculum size and time of observation, following simulations of our alternative model. Infection success again increases overall with the inoculum size, and, in general, is higher when observed sooner. Note, however, the non-monotonic, double-peaked profile of infection success versus inoculum for observation times of approximately 10–16 days.
(TIF)

S1 Code Supplementary Online Material which includes all code necessary for the reported analyses and figures. (PDF)
