# Supplementary figures and images for: Dose-dependent interaction of parasites with tiers of host defense predicts “wormholes” that prolong infection at intermediate inoculum sizes
Source: PLoS Comput Biol. 2024 Dec 6;20(12):e1012652. doi: 10.1371/journal.pcbi.1012652 (PMC11654943; doi:10.1371/journal.pcbi.1012652)

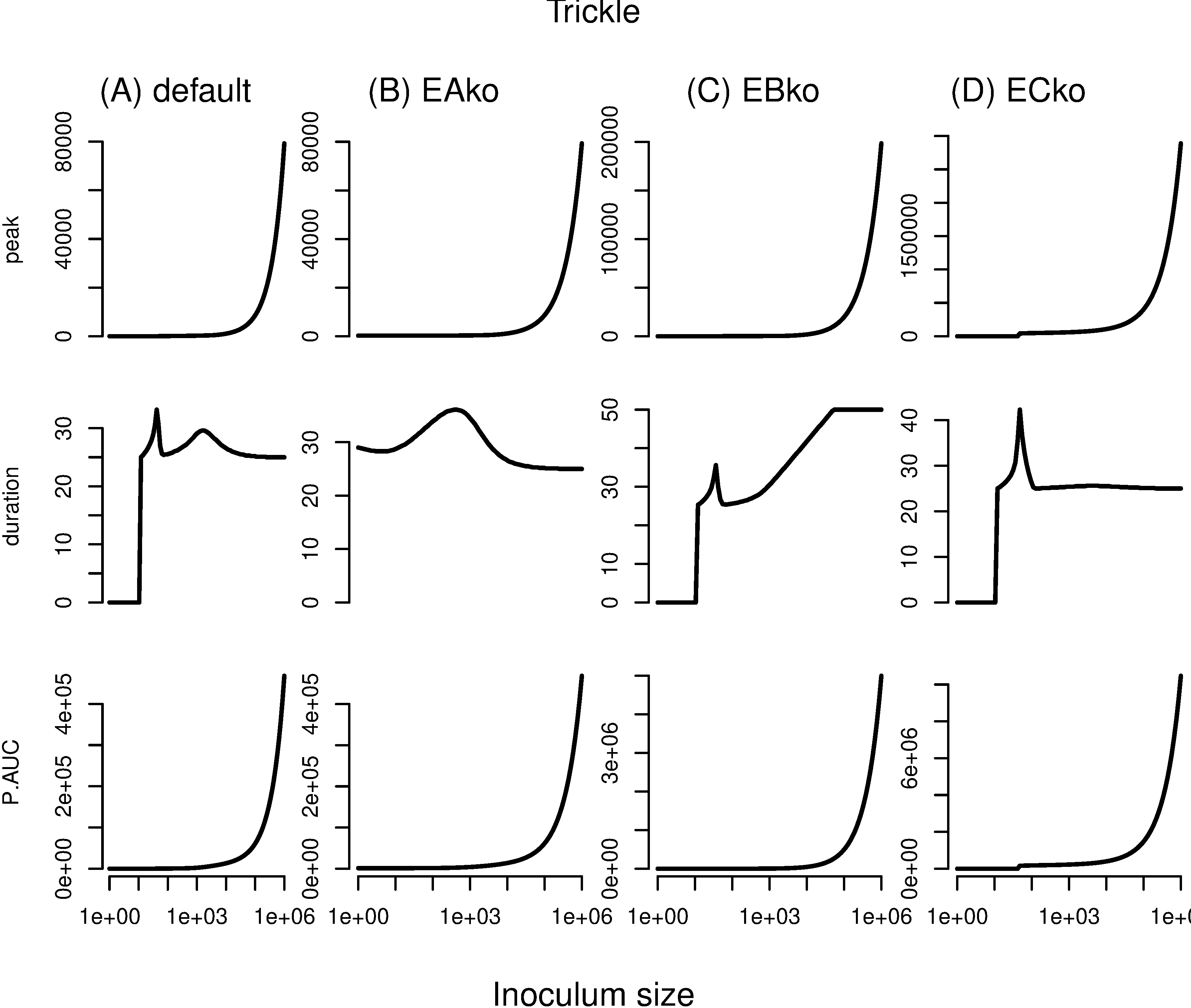

Supplement: S1 Fig — Peak parasite load (top row), infection duration (middle row), and cumulative parasite load (AUC, or area under the curve; bottom row) statistics for deterministic simulations across a range of inoculum sizes in 4 host types: (A) wildtype (“default”), (B) barrier knockouts (“EAko”), (C) second-tier knockouts (“EBko”), and (D) third-tier knockouts (“ECko”). Here, all doses arrive slowly over time, in a trickle rather than bolus inoculation. (TIF) [file pcbi.1012652.s001.tif]

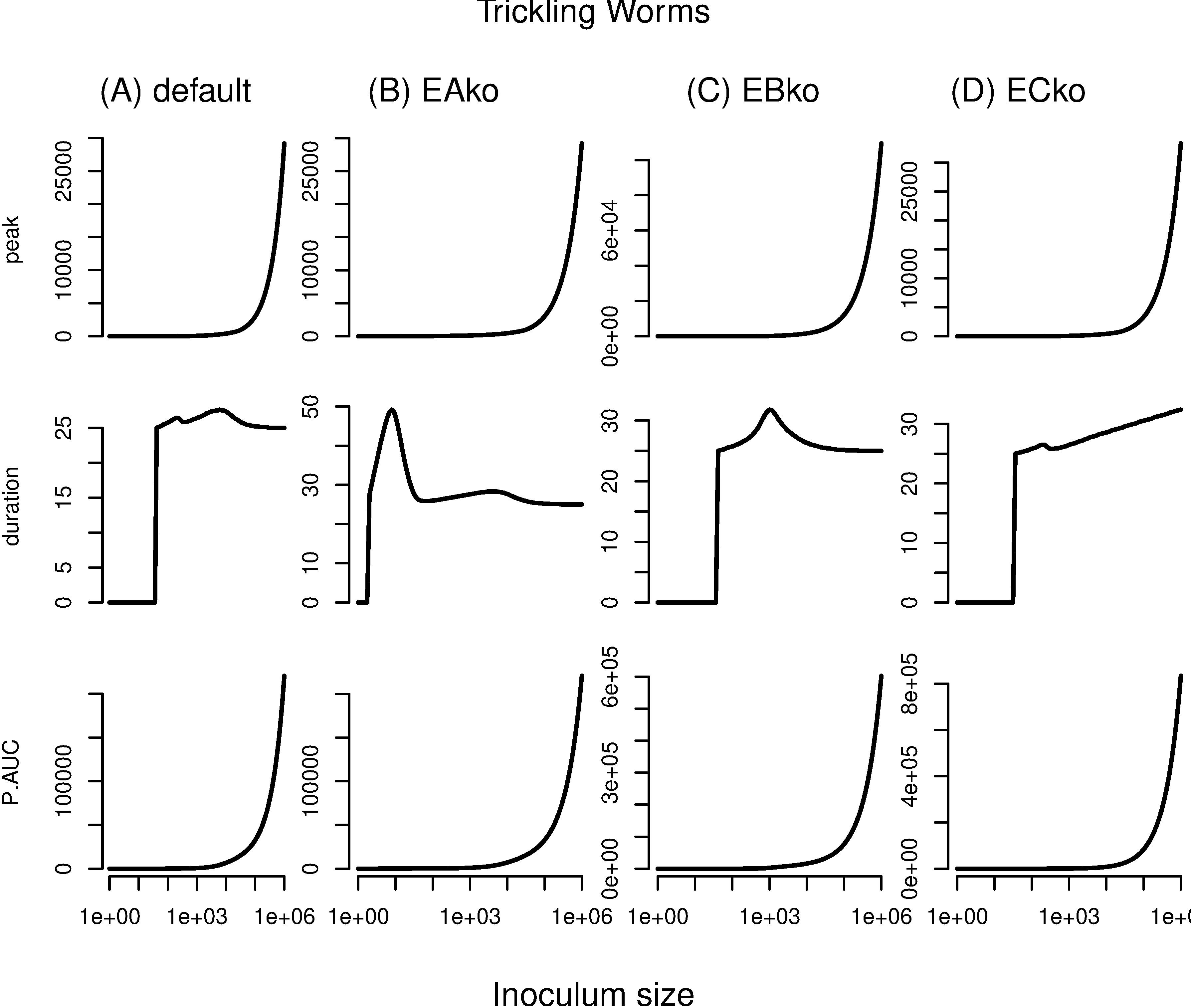

Supplement: S2 Fig — Peak parasite load (top row), infection duration (middle row), and cumulative parasite load (AUC, or area under the curve; bottom row) statistics for deterministic simulations across a range of inoculum sizes in 4 host types: (A) wildtype (“default”), (B) barrier knockouts (“EAko”), (C) second-tier knockouts (“EBko”), and (D) third-tier knockouts (“ECko”). Here, all parasites are non-replicating macroparasites/worms, that arrive slowly in a trickle inoculation. (TIF) [file pcbi.1012652.s002.tif]

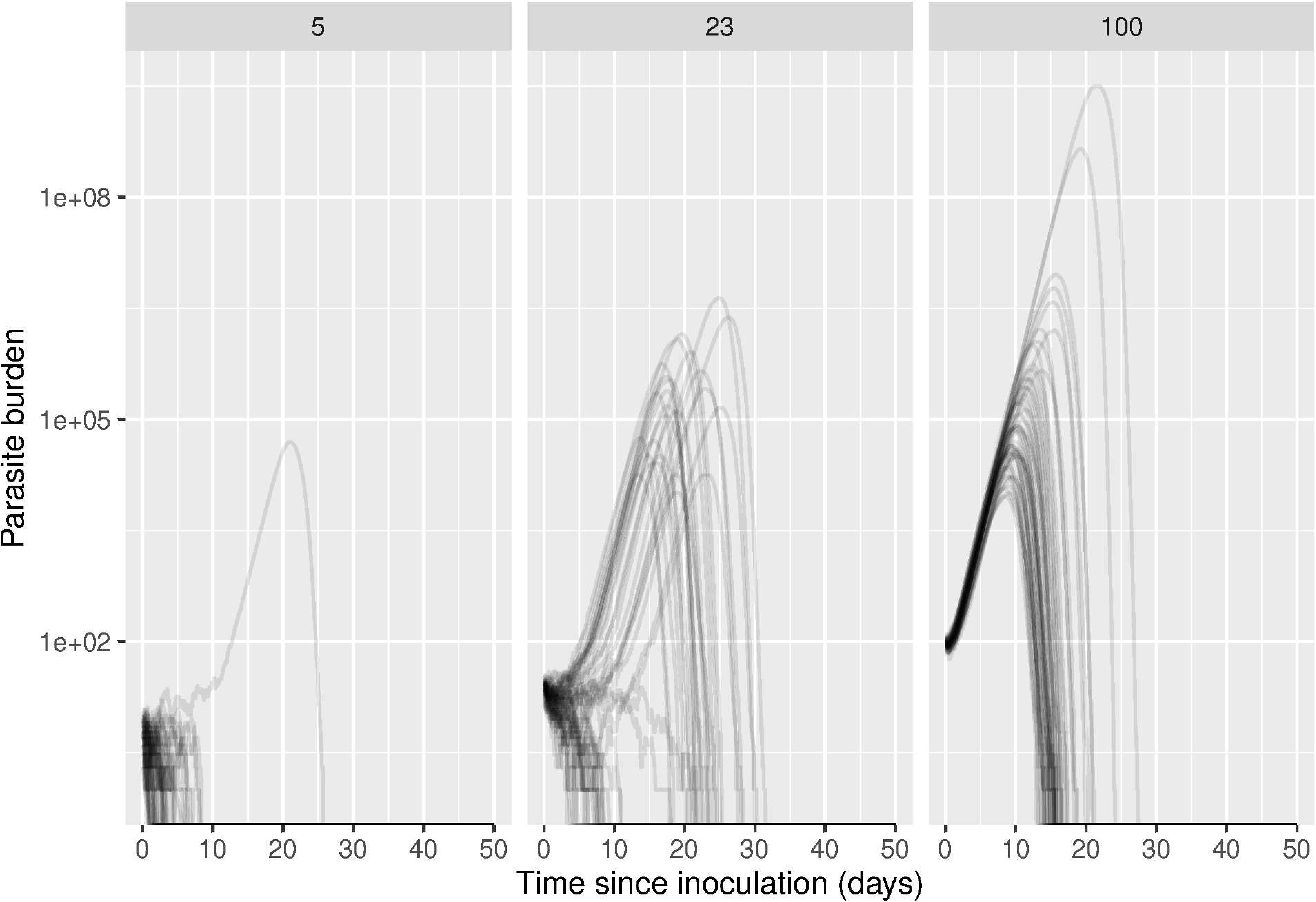

Supplement: S3 Fig — (TIF) [file pcbi.1012652.s003.tif]

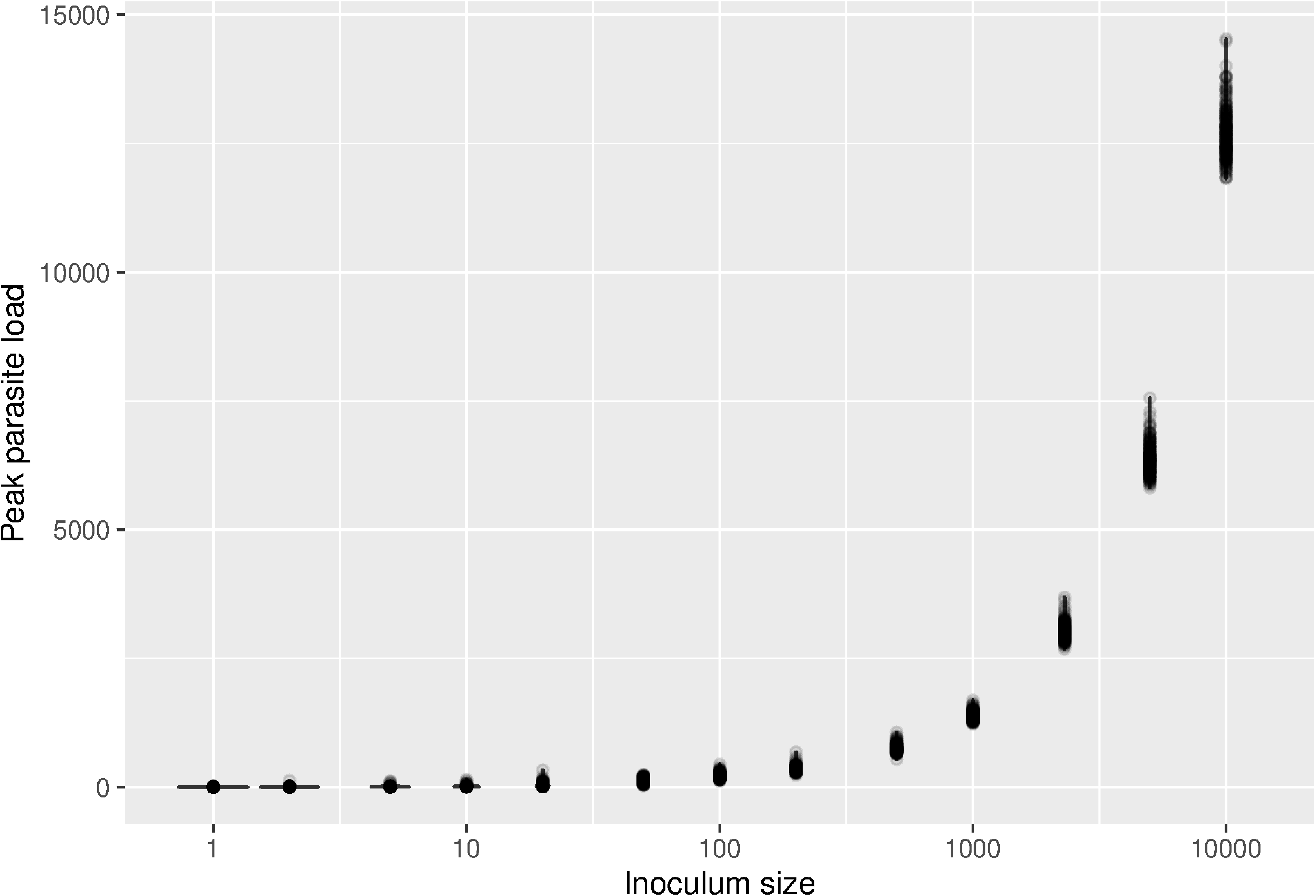

Supplement: S4 Fig — (TIF) [file pcbi.1012652.s004.tif]

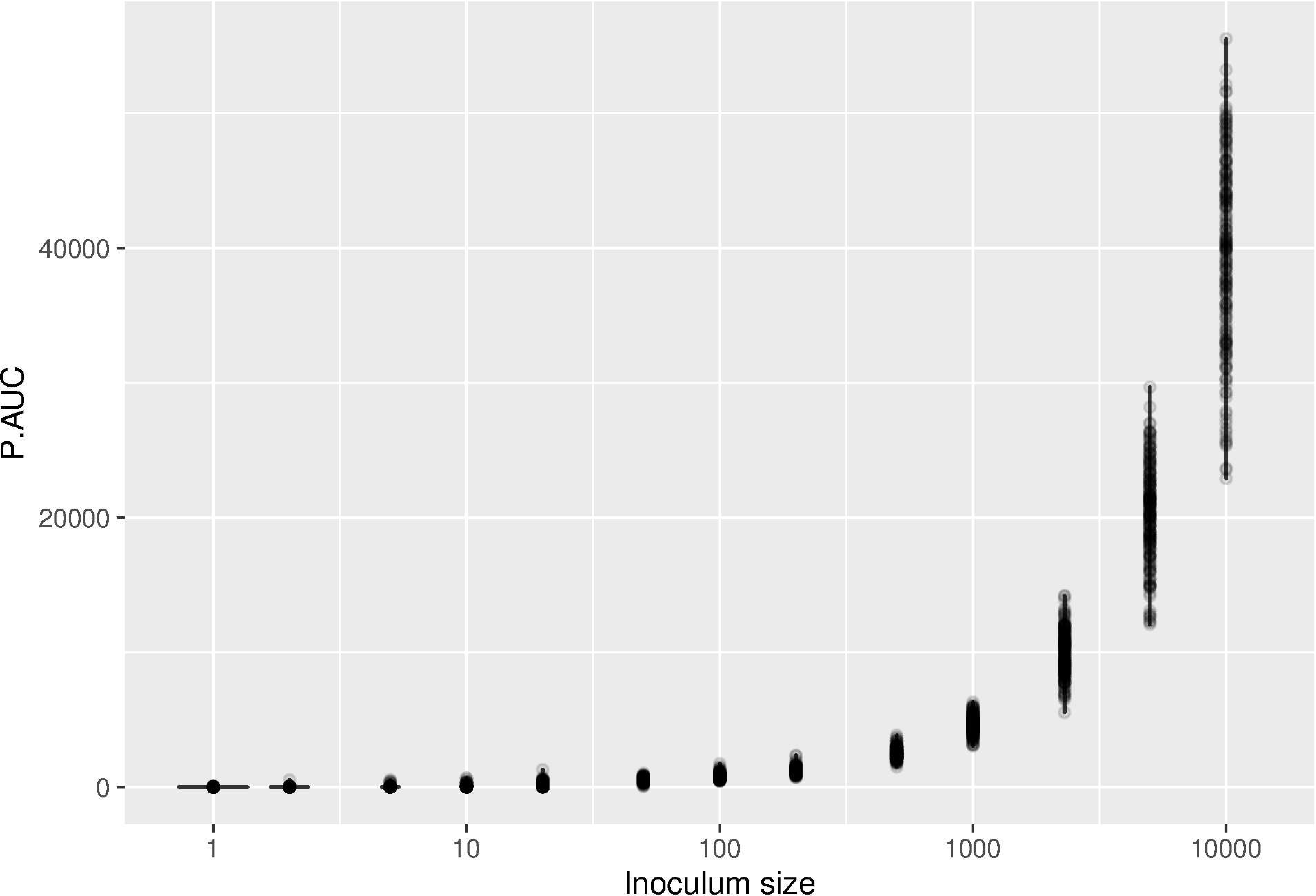

Supplement: S5 Fig — (TIF) [file pcbi.1012652.s005.tif]

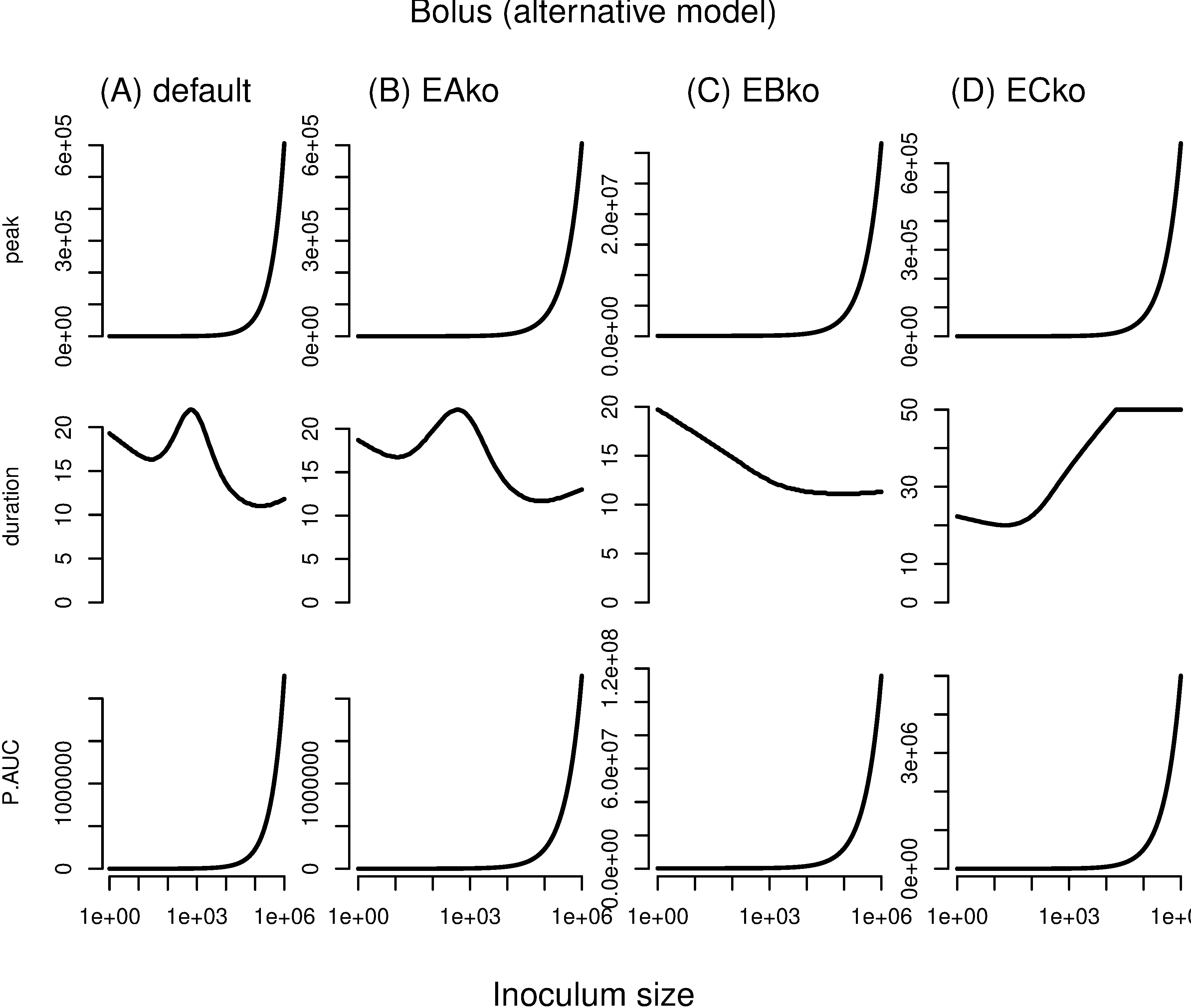

Supplement: S6 Fig — Peak parasite load (top row), infection duration (middle row), and cumulative parasite load (AUC, or area under the curve; bottom row) statistics for deterministic simulations in our alternative model, across a range of inoculum sizes in 4 host types: (A) wildtype (“default”), (B) barrier knockouts (“EAko”), (C) second-tier knockouts (“EBko”), and (D) third-tier knockouts (“ECko”). Here, all parasites are microparasites that arrive all at once in a bolus. (TIF) [file pcbi.1012652.s006.tif]

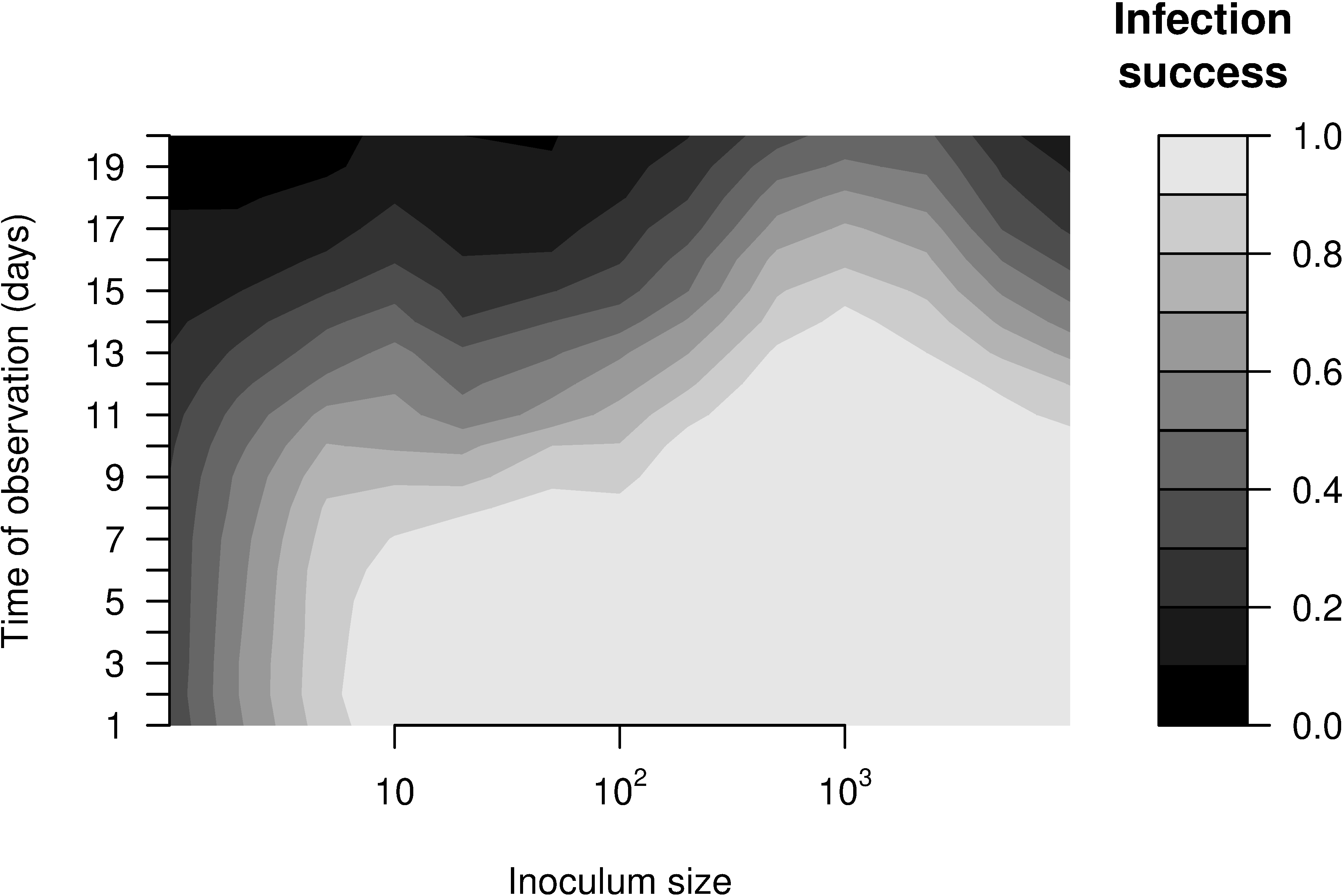

Supplement: S7 Fig — Infection success again increases overall with the inoculum size, and, in general, is higher when observed sooner. Note, however, the non-monotonic, double-peaked profile of infection success versus inoculum for observation times of approximately 10–16 days. (TIF) [file pcbi.1012652.s007.tif]
