# Supplementary material for: Dose-dependent interaction of parasites with tiers of host defense predicts “wormholes” that prolong infection at intermediate inoculum sizes
Source: PLoS Comput Biol. 2024 Dec 6;20(12):e1012652. doi: 10.1371/journal.pcbi.1012652 (PMC11654943; doi:10.1371/journal.pcbi.1012652)

Dose-dependent interaction of parasites with tiers of host defense generates “wormholes” that prolong infection at intermediate inoculum sizes - Supplementary Online Material

Andrea L. Graham and Roland R. Regoes

November 26, 2024

Technical preliminaries

Load required packages:

```r
library(deSolve, quietly = TRUE)
library(ggplot2, quietly = TRUE)
library(magrittr, quietly = TRUE)
library(plyr, quietly = TRUE)
library(reshape2, quietly = TRUE)
```

Model with exposure schedule

The function below implements the model we used in this study (Eqn 1-4 in the main text). In addition to the variables and parameters, we defined in the main text, we define variables for the dosing schedule (`exposure`), the time after exposure when the simulation is terminated (`tmax`), and a variable `deterministic` to switch from detemerinistic to stochastic simulations.

```r
dose.model <- function(exposure=exp.0,
                       tmax=30,
                       params=pars.0,
                       deterministic=T){

    ## exposure rate function
    eta.1 <- function(t, e){
        with(as.list(e),{
            if(any(schedule <= t & t < schedule+duration))
                out <- max.dosing.rate
            else out <- 0
            return(out)
        })
    }
    eta <- function(t.vec, e=exposure) {
        sapply(t.vec, function(t) eta.1(t, e=e))
    }

    if(deterministic){
        ## define derivatives for ODEs:
        derivs <- function(t,x,p){
```

```
        P <- x[1]
        EA <- x[2]
        EB <- x[3]
        EC <- x[4]
        with(as.list(p),{
            dP  <- eta(t) + P*r  - P*(gammaA*EA + gammaB*EB + gammaC*EC)
            dEA <- sigmaA*(1-EA/EA0) - gammaA*P*EA
            dEB <- sigmaB*(1 - EB/KEB)*EB*P/(hB+P)
            dEC <- sigmaC*(1 - EC/KEC)*EC*P/(hC+P)

            list(c(dP, dEA, dEB, dEC))
        })
    }

    ## solve ODEs:
    require(deSolve)
    P.init <- 0
    inits <- c(P=P.init, EA=params[["EA0"]],
               EB=params[["EB0"]], EC=params[["EC0"]])
    times <- seq(0, tmax, length=501)
    output <-as.data.frame(lsoda(inits, times, derivs, params))
}

if(!deterministic){
    require(adaptivetau)
    P.init <- 0
    inits <- c(P=P.init, EA=params[["EA0"]],
               EB=params[["EB0"]], EC=params[["EC0"]])

    transitions <-
        ssa.maketrans(c("P", "EA", "EB", "EC"),
                      rbind("P", +1), # growthP
                      rbind("P", -1, "EA", -1), # deathP and EA
                                    # by killing interaction
                      rbind("P", -1), # deathP by killing interaction
                      rbind("P", -1), # deathP by killing interaction
                      rbind("EA", +1), # replenishmentEA
                      rbind("EB", +1), # growthEB
                      rbind("EB", -1), # deathEB
                      rbind("EC", +1), # growthEC
                      rbind("EC", -1)) # deathEC

    trans.rates <- function(x, p, t){
        P <- x[1]
        EA <- x[2]
        EB <- x[3]
        EC <- x[4]
        with(as.list(p),{
            rates <-
                c(eta(t) + P * r,
                  gammaA*P*EA,
                  gammaB*P*EB,
                  gammaC*P*EC,
```

```
                      sigmaA*(1-EA/EA0),
                      sigmaB*EB*P/(hB+P),
                      sigmaB*EB*P/(hB+P)*EB/KEB, # "logistic growth"-death
                      sigmaC*EC*P/(hC+P),
                      sigmaC*EC*P/(hC+P)*EC/KEC)
                names(rates) <- NULL
                return(rates)
            })
        }

        output <-
            as.data.frame(ssa.adaptivetau(inits, transitions,
                                          trans.rates, params,
                                          tf=tmax))
    }

    ## print output:
    output
}
```

The default parameters are defined as:

```
pars.0 <- c(r=1, gammaA=0.015, gammaB=0.00012, gammaC=0.05,
            sigmaA=30, sigmaB=40, sigmaC=0.5, hB=1000, hC=1000, KEB=1e4, KEC=1e6,
            EA0=100, EB0=1, EC0=1)
```

See the main text for their definition and the justification of their default values.

The variable `exposure` captures the dosing schedule in terms of the intensity of each dosing `max.dosing.rate`, its `duration`, and the times, `schedule`, at which they occur. The default exposure in our model is a short, one-hour pulse of the parasite, which we refer to as "bolus" inoculation.

```
t.bolus <- 1/24
exp.0 <- list(max.dosing.rate=100/t.bolus, duration=t.bolus, schedule=c(0))
```

This one-hour `duration` together with the `max.dosing.rate` yield an inoculum size of 100.

To run this model with default parameters simply execute in R:

```
sim.1 <- dose.model()
```

Systematic exploration of the infection dynamics across inoculum doses and schedules

Time courses (Figure 1A)

To generate figures similar to Figure 1A, we implemented the plotting function that draws time course of all four variables for multiple inocula:

```
plot.dose.schedule.field <- function(inocula=c(5, 10, 20, 50, 100, 200, 500, 1000, 2000, 5000),
                                     t.max=15,
                                     schedule.type="bolus",  #"trickle"
                                     params.0=pars.0,
                                     deterministic=T){

    require(ggplot2)
```

```
    sims <- vector(mode="list", length=length(inocula))
    for(i in seq_along(inocula)){
        if(schedule.type=="bolus"){
            e <- list(max.dosing.rate = inocula[[i]]/t.bolus,
                      duration = t.bolus, schedule = c(0))
        }
        if(schedule.type=="trickle"){
            e <- list(max.dosing.rate = inocula[[i]]/(t.max/2),
                      duration = t.max/2, schedule = c(0))
        }
        sims[[i]] <-
            dose.model(exposure = e,
                       tmax = t.max,
                       params = params.0,
                       deterministic = deterministic)
    }
    names(sims) <- as.character(inocula)

    require(magrittr)
    require(plyr)
    require(reshape2)

    variable.descr <- c(P="Parasite\nload, P",
                        EA="Barrier\ndefense, EA",
                        EB="Innate\ndefense, EB",
                        EC="Adaptive\ndefense, EC")

    sims %>% ldply(.id="inoculum") %>%
        subset(select=c(time,inoculum,P,EA,EB,EC)) %>%
        melt(id=c("time","inoculum")) %>%

        ggplot(aes(x=time, y=value,
                   group=inoculum,
                   size=inoculum,
                   linetype=inoculum,
                   color=inoculum))+
        facet_grid(variable~., labeller = as_labeller(variable.descr),
                   switch = "y", scales="free_y")+
        geom_line() +
        scale_x_continuous("Time since inoculation (days)", limits=c(0,t.max)) +
        scale_y_log10("", limits=c(1,1.25e4)) +
        scale_color_manual(values=sapply(0:(length(inocula)-1),
                                         function(x) rgb(x/(length(inocula)-1),
                                                         0.6*x/(length(inocula)-1),
                                                         0))) +
#        scale_color_manual(values=sapply(1:length(inocula),
#                                         function(x) rgb(x/length(inocula),
#                                                         0,
#                                                         1-x/length(inocula)))) +
        theme_bw() -> graph

    ## output figure
    return(graph)
```

```
}
```

To run this function and generate Figure 1A execute:

```
plot.dose.schedule.field(inocula=c(10, 20, 50, 100, 500, 2000, 10000),
                         t.max=15,
                         schedule.type="bolus",
                         params.0=pars.0) -> fig1

## highlight the longest and shortest durations:
#fig1 <- fig1 + scale_size_manual(values = c(0.3,1,0.3,0.7,0.3,1,0.3))
#fig1 <- fig1 + scale_size_manual(values = rep(0.5,7)) +
#    scale_linetype_manual(values=c(2,1,2,1,2,1,2))
fig1 <- fig1 + scale_size_manual(values = c(0.3,1,0.3,0.7,0.3,1,0.3)) +
    scale_linetype_manual(values=rep(1,7))

print(fig1)
```

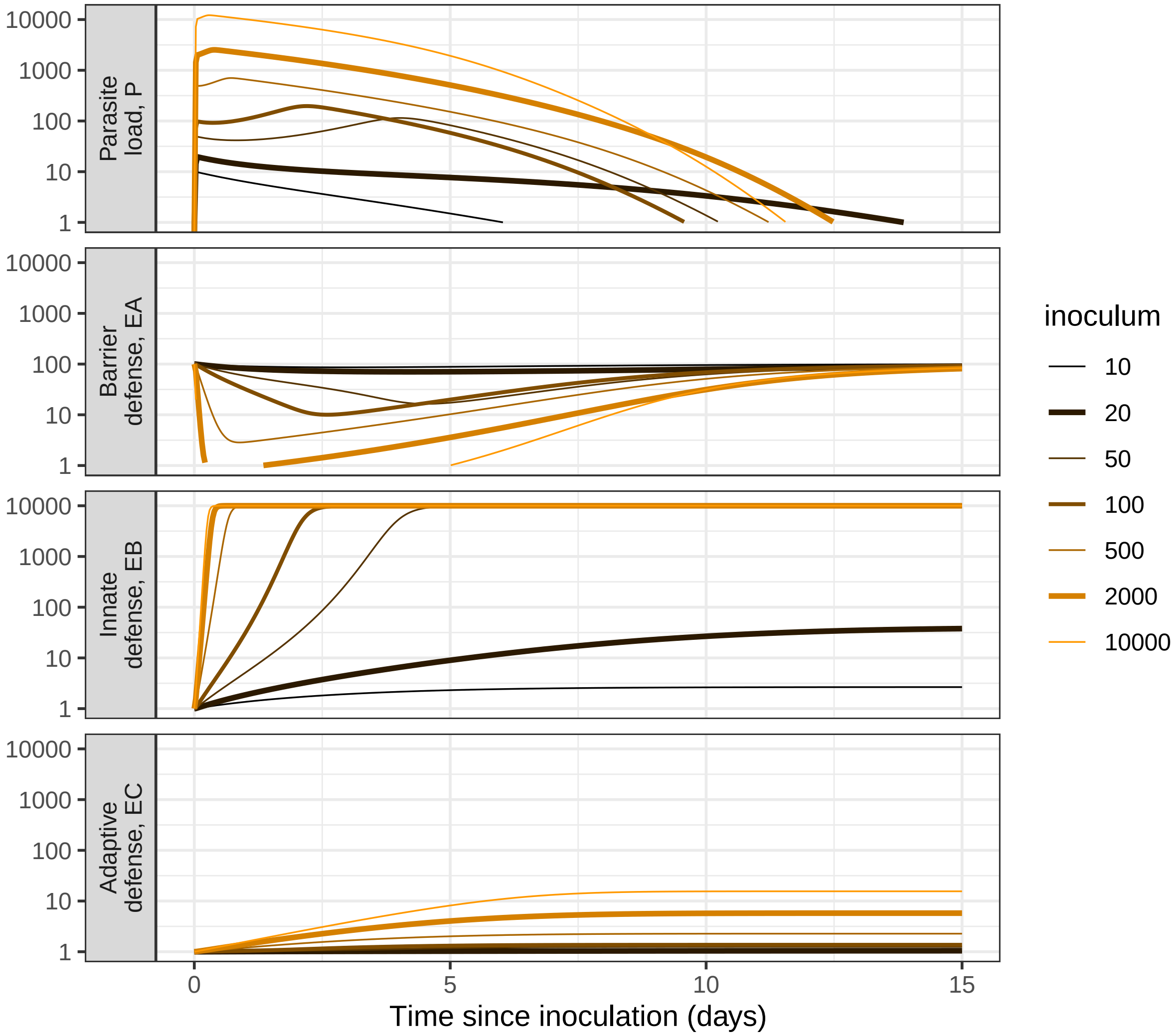

Time courses of the parasite load for varying doses as a contour plot (Figure 1B)

To illustrate the multiple turning points of infection duration as a function of the inoculum, we plot the parasite load `P` versus time and inoculum dose as a contour plot.

```
plot.P.time.dose.contour <- function(inocula=10^seq(0,6,length=201),
                                     t.max=20,
                                     schedule.type="bolus",  #"trickle"
                                     params.0=pars.0,
                                     deterministic=T){

    t.seq <- dose.model(tmax=t.max)$time
    P.matrix <- matrix(NA,
                       ncol=length(t.seq), nrow=length(inocula),
                       byrow=T)
    for(i in seq_along(inocula)){
        if(schedule.type=="bolus"){
            e <- list(max.dosing.rate = inocula[[i]]/t.bolus,
                      duration = t.bolus, schedule = c(0))
        }
        if(schedule.type=="trickle"){
            e <- list(max.dosing.rate = inocula[[i]]/(t.max/2),
                      duration = t.max/2, schedule = c(0))
        }
        P.matrix[i,] <-
            dose.model(exposure = e,
                       tmax = t.max,
                       params = params.0,
                       deterministic = deterministic)$P
        ## to let small values disappear from plot
        P.matrix[i,] <- replace(P.matrix[i,], P.matrix[i,]<1,NA)
    }

    filled.contour(x=log10(inocula),
                   y=t.seq,
                   z=log10(P.matrix),
                   ylim=range(t.seq),
                   plot.axes={
                       axis(1, at=c(0, 1, log10(23), 2, log10(2000), 4),
                            labels=expression(1, 10, 23, 100, 2000, 10000),
                            las=1)
                       axis(2)
                       abline(v=log10(23), lty=2)
                       abline(v=log10(2000), lty=2)
                   },
                   key.title=title(main="Parasite\nload, P", cex.main=0.9),
                   key.axes=axis(4, at=0:4,
                                 labels=expression(1, 10, 10^2, 10^3, 10^4),
                                 las=2),
                   xlab="Inoculum size",
                   ylab="Time since inoculation (days)")
}

plot.P.time.dose.contour(inocula=10^seq(0, 4, length=201),
                         t.max=20,
```

```
                          schedule.type="bolus",
                          params.0=pars.0)
```

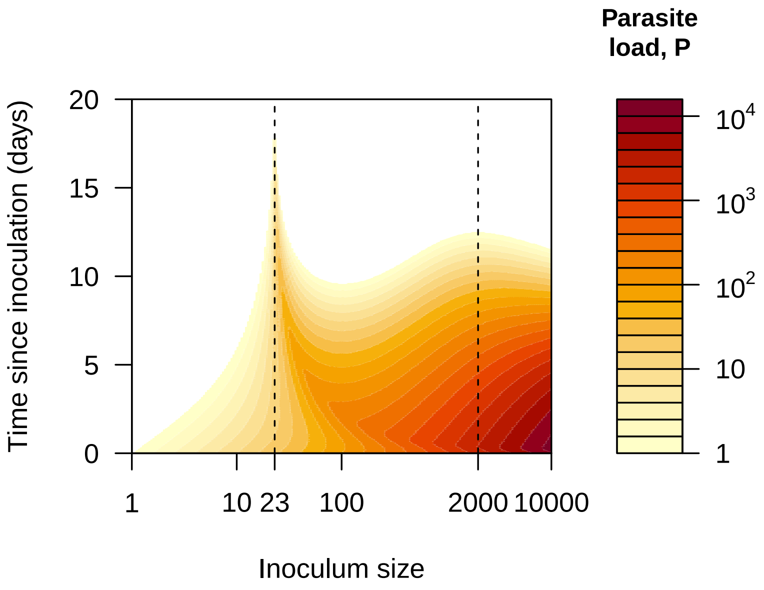

Figure 1C requires algorithms used for Figure 2, and therefore appears after Figure 2 below.

Infection summary statistics across inocula (Figure 2)

Here we list the functions needed to plot infection statistics across different inoculum doses and exposure types. As exposure types we consider a single inoculation with a single dose (`"bolus"`), or continuous doses for a period of time (`"trickle"`).

The following function extract a statistics from a simulation.

```
extract.stats <- function(sim=dose.model(exposure=exp.0),
                          what="peak"){
    last <- dim(sim)[1]

    if(what=="peak") return(max(sim$P, na.rm=T))
    if(what=="Tpeak") return(sim$time[sim$P==max(sim$P, na.rm=T)][1])
    if(what=="duration") {
        if(any(sim$P>=1, na.rm=T)) return(max(sim$time[sim$P>=1], na.rm=T))
        else return(0)
    }
    if(what=="P.AUC") {
        return(sum(sim$P[-last]*diff(sim$t)))
    }
    if(what=="EC.final") return(sim$EC[last])
}
```

The statistics can be specified as an argument to the variable `what`. It can take the arguments:

- `"peak"` - the peak parasite load during the infection
- `"Tpeak"` - the timing of this peak
- `"duration"` - the overall duration of the infection
- `"P.AUC"` - the area under the parasite load curve, a measure of the overall production of parasites
- `"EC.final"` - the final level of the adaptive immune response

The following function runs simulations for a range of inocula and inoculation types and extracts the infection statistics:

```
stats.vs.dose <- function(model="dose.model", # or "alt.dose.model"
                          stats=c("peak", "Tpeak", "duration", "P.AUC", "EC.final"),
                          inocula=c(10,100,1000),
                          schedule.type="bolus",
                          t.max=50,
                          params.0=pars.0){

    if(model=="dose.model") dm <- dose.model
    if(model=="alt.dose.model") dm <- alt.dose.model

    output <- data.frame(inoculum=inocula)
    for(s in stats) output <- cbind(output, NA)
    dimnames(output)[[2]][-1] <- stats
    attr(output, "parameters") <- params.0
    attr(output, "schedule.type") <- schedule.type
    attr(output, "t.max") <- t.max

    for(i in inocula){
        if(schedule.type=="bolus")
            e <- list(max.dosing.rate=i/t.bolus,
                      duration=t.bolus,
                      schedule=c(0))
        if(schedule.type=="trickle")
            e <- list(max.dosing.rate=i/(t.max/2),
                      duration=t.max/2,
                      schedule=c(0))
        sim.i <- dm(exposure=e,
                    tmax=t.max,
                    params=params.0,
                    deterministic=T)
        for(s in stats) {
            output[output$inoculum==i, s] <-
                extract.stats(sim=sim.i,
                              what=s)
        }
    }

    output
}
```

For bolus inoculations with default parameters and parameters describing immune knockouts, we obtain:

```
inoc.seq <- 10^seq(0,6,length.out=101)
svd.0 <-
    stats.vs.dose(inocula=inoc.seq,
                  params.0=pars.0)

pars.EAko <- pars.0
pars.EAko[c("gammaA","EA0")] <- c(0.0,1e-3)
svd.EAko <-
    stats.vs.dose(inocula=inoc.seq,
                  params.0=pars.EAko)
```

```
pars.EBko <- pars.0
pars.EBko[c("gammaB","EB0")] <- c(0.0,0.0)
svd.EBko <-
    stats.vs.dose(inocula=inoc.seq,
                  params.0=pars.EBko)

pars.ECko <- pars.0
pars.ECko[c("gammaC","EC0")] <- c(0.0,0.0)
svd.ECko <-
    stats.vs.dose(inocula=inoc.seq,
                  params.0=pars.ECko)
```

The following plots these statistics by knockout:

```
par(mfcol=c(3,4), omi=c(1,1,1,0), mar=c(1, 4, 1, 0) + 0.1, xpd=T)->op
for(svd in list(svd.0, svd.EAko, svd.EBko, svd.ECko)){
    for(s in c("peak", "duration", "P.AUC")){
        if(any(!svd==svd.0)) yl<-"" else yl<-s
        plot(svd$inoculum, svd[,s],
             type="l", lwd=2, log="x",
             main="", axes=F,
             ylim=c(0,max(1,max(svd[,s]))),
             xlab="", ylab=yl)
        axis(2)
    }
    par(mar=c(1, 2, 1, 0) + 0.1)
    axis(1)
}
rm(svd,s,yl)
mtext("(A) default", adj=0.1, outer=T)
mtext("(B) EAko", adj=0.35, outer=T)
mtext("(C) EBko", adj=0.65, outer=T)
mtext("(D) ECko", adj=0.9, outer=T)
mtext("Inoculum size", adj=0.5, side=1, outer=T, line=3)
mtext("Bolus", adj=0.5, side=3, outer=T, line=3)
```

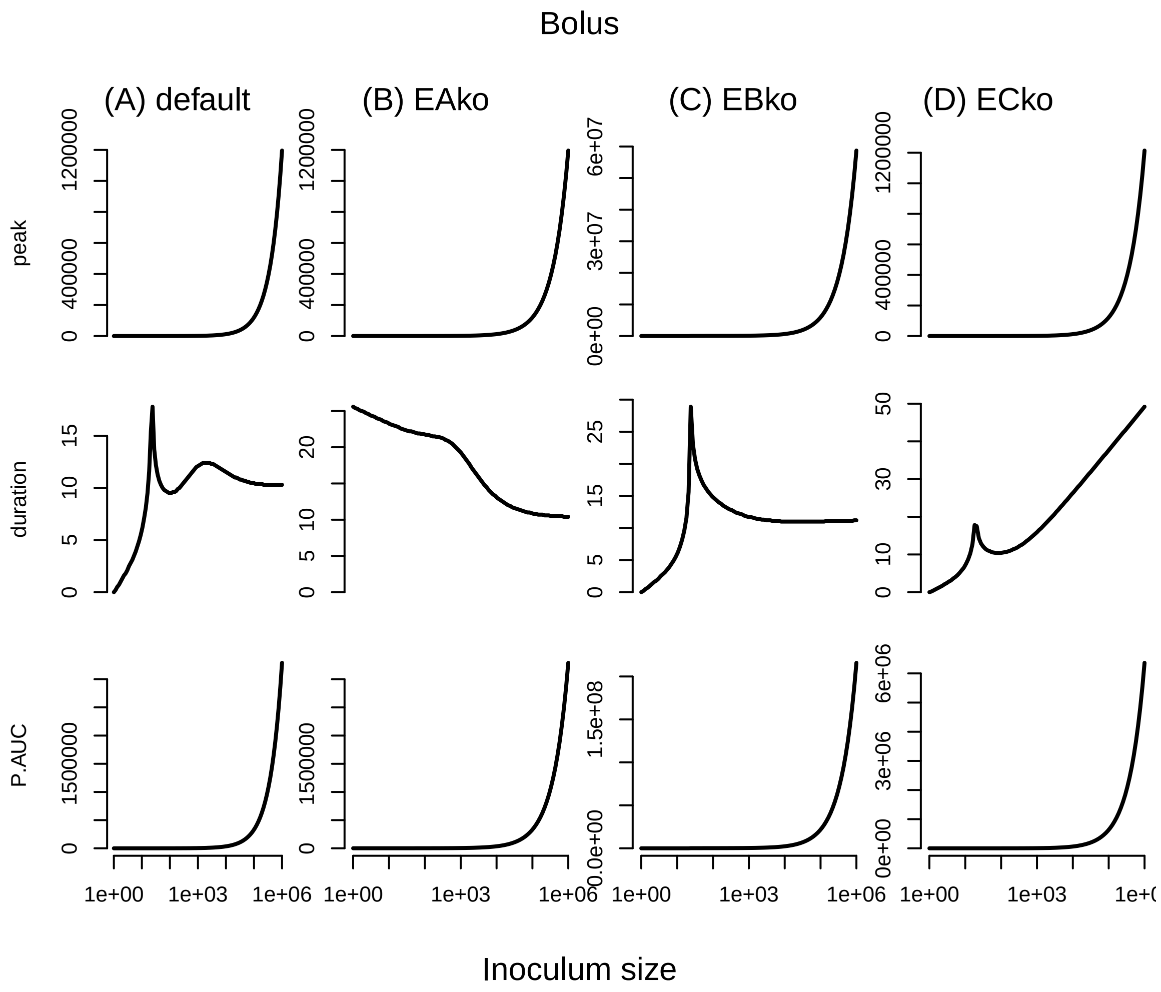


```
par(op);rm(op)
```

Infection duration vs strength of the first and second tier of the immune response (Figure 1C)

How does the duration change for different strength of the first and second tier of the immune response?

```
svd.strength <- stats.vs.dose(stats=c("duration"),
                              inocula=10^seq(0,6,length.out=201), #inoc.seq,
                              params.0=pars.0)
svd.strength <- cbind(svd.strength, par.set="pars.0", f=1)

#f.seq <- c(0.3, 0.4, 0.43, 0.46, 0.5, 0.6, 0.7, 0.8, 0.9, 1.1, 1.2, 1.5)
f.seq <- c(0.3, 0.4, 0.5, 0.6, 0.8, 1.5)
nfs <- length(f.seq)

pars.col <- vector("character", length=nfs)
pars.col[1] <- "grey"

for(i in 1:nfs){
    pars.col[i+1]<-rgb(1-(i-1)/nfs,0.6*(1-(i-1)/nfs),0,alpha=1)
    p <- pars.0
    f <- f.seq[i]
```

```
    p["gammaA"] <- p["gammaA"]*f
    p["gammaB"] <- p["gammaB"]*f
    svd.strength <-
        rbind(svd.strength,
              cbind(stats.vs.dose(stats=c("duration"),
                                  inocula=inoc.seq,
                                  params.0=p),
                    par.set=paste0("pars.",formatC(i, width = 2,
                                                    format = "d",
                                                    flag = "0")),
                    f=f))
}
svd.strength$f <- factor(svd.strength$f)
rm(p,f,i)

#ggplot(data=svd.strength, aes(x=inoculum, y=duration, colour=par.set, show.legend)) +
ggplot(data=svd.strength, aes(x=inoculum, y=duration, colour=f, show.legend)) +
    geom_line() +
    scale_x_log10("Inoculum size",
                  breaks=c(1e1,1e3,1e5),
                  labels=expression(10, 10^3, 10^5)) +
    scale_y_continuous("Infection duration") +
    labs(colour=expression(atop("Factor scaling", gamma[A]*" and "*gamma[B]))) +
    scale_color_manual(values=pars.col[c(2:6,1,7)],
                       labels=c(as.character(f.seq)[1:5],
                                "1 (default)",
                                as.character(f.seq)[6]))
```

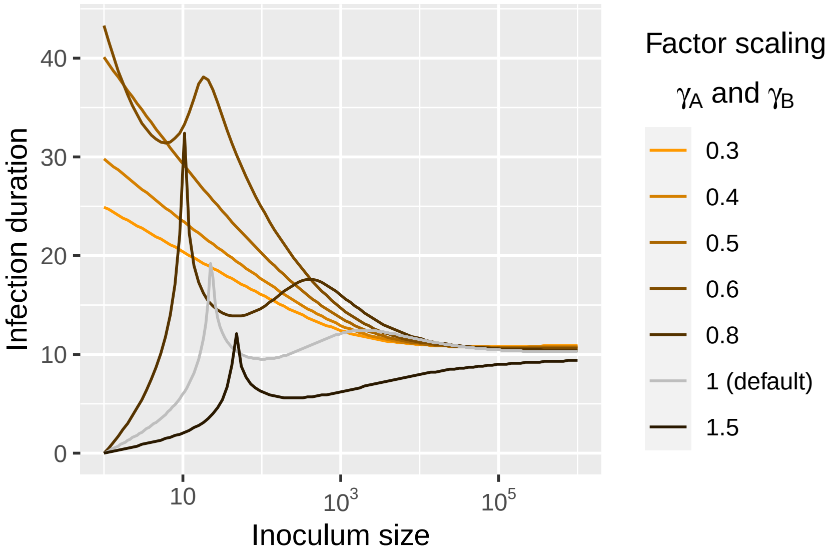

Infection summary statistics across inocula for non-replicating parasites (Figure 3), and for trickle exposure (Figures S1, and S2)

For bolus inoculations for non-replicating parasites (“worms”), and parameters describing immune knockouts, we obtain:

```
pars.0.w <- pars.0
pars.0.w["r"] <- 0.0
```

```r
svd.0.w <-
    stats.vs.dose(inocula=inoc.seq,
                  params.0=pars.0.w)

pars.EAko.w <- pars.EAko
pars.EAko.w["r"] <- 0.0
svd.EAko.w <-
    stats.vs.dose(inocula=inoc.seq,
                  params.0=pars.EAko.w)

pars.EBko.w <- pars.EBko
pars.EBko.w["r"] <- 0.0
svd.EBko.w <-
    stats.vs.dose(inocula=inoc.seq,
                  params.0=pars.EBko.w)

pars.ECko.w <- pars.ECko
pars.ECko.w["r"] <- 0.0
svd.ECko.w <-
    stats.vs.dose(inocula=inoc.seq,
                  params.0=pars.ECko.w)
```

The following plots these statistics by knockout:

```r
par(mfcol=c(3,4), omi=c(1,1,1,0), mar=c(1, 4, 1, 0) + 0.1, xpd=T)->op
for(svd in list(svd.0.w, svd.EAko.w, svd.EBko.w, svd.ECko.w)){
    for(s in c("peak", "duration", "P.AUC")){
        if(any(!svd==svd.0.w)) yl<-"" else yl<-s
        plot(svd$inoculum, svd[,s],
             type="l", lwd=2, log="x",
             main="", axes=F,
             ylim=c(0,max(1,max(svd[,s]))),
             xlab="", ylab=yl)
        axis(2)
    }
    par(mar=c(1, 2, 1, 0) + 0.1)
    axis(1)
}
rm(svd,s,yl)
mtext("(A) default", adj=0.1, outer=T)
mtext("(B) EAko", adj=0.35, outer=T)
mtext("(C) EBko", adj=0.65, outer=T)
mtext("(D) ECko", adj=0.9, outer=T)
mtext("Inoculum size", adj=0.5, side=1, outer=T, line=3)
mtext("Worms", adj=0.5, side=3, outer=T, line=3)
```

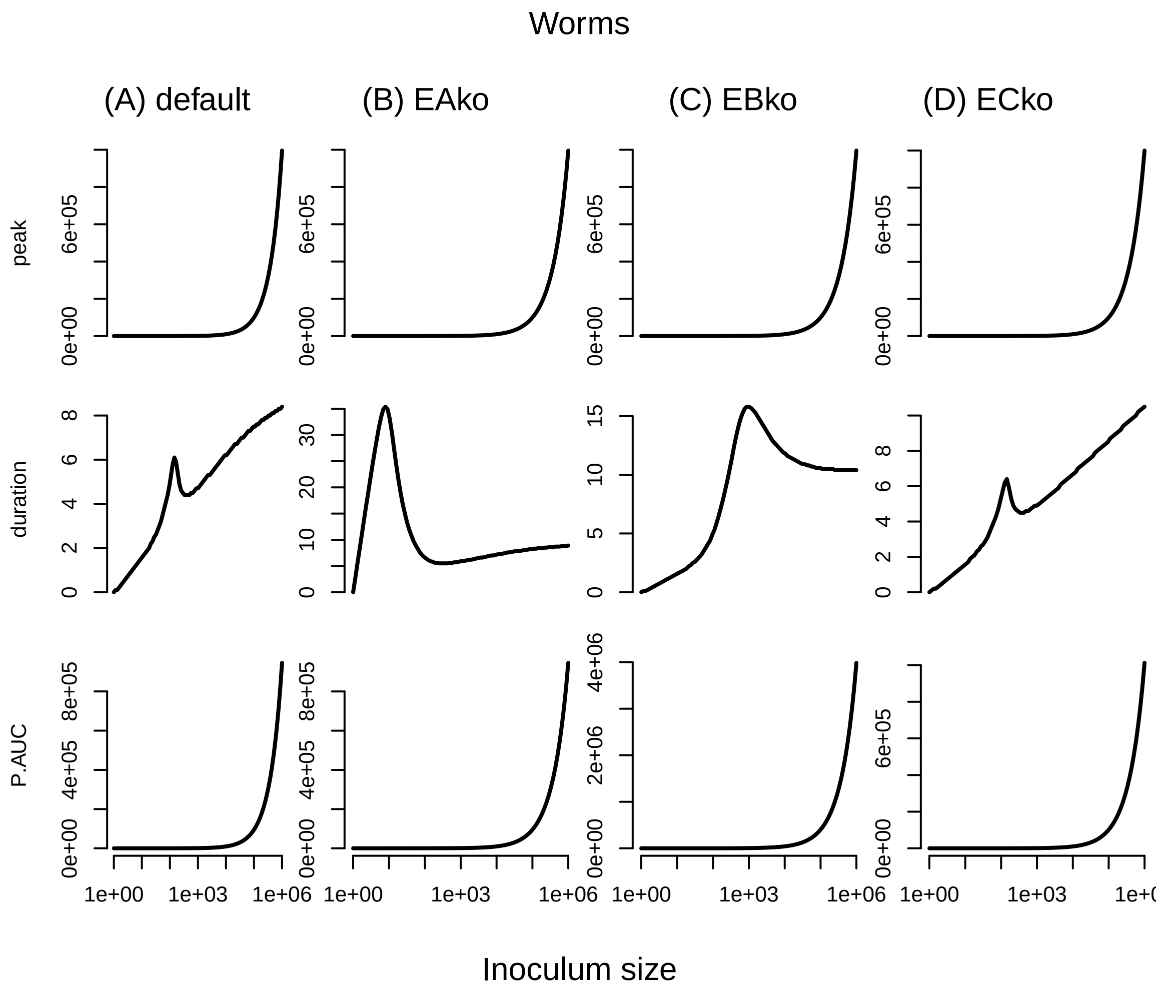


```
par(op);rm(op)
```

For continuous ("trickle") inoculations for a parasite with default parameters and parameters describing immune knockouts, we obtain:

```
svd.0.t <-
    stats.vs.dose(inocula=inoc.seq,
                  schedule.type="trickle",
                  params.0=pars.0)
svd.EAko.t <-
    stats.vs.dose(inocula=inoc.seq,
                  schedule.type="trickle",
                  params.0=pars.EAko)
svd.ECko.t <-
    stats.vs.dose(inocula=inoc.seq,
                  schedule.type="trickle",
                  params.0=pars.EBko)
svd.EBko.t <-
    stats.vs.dose(inocula=inoc.seq,
                  schedule.type="trickle",
                  params.0=pars.ECko)
```

The following plots these statistics by knockout:

```
par(mfcol=c(3,4), omi=c(1,1,1,0), mar=c(1, 4, 1, 0) + 0.1, xpd=T)->op
for(svd in list(svd.0.t, svd.EAko.t, svd.EBko.t, svd.ECko.t)){
    for(s in c("peak", "duration", "P.AUC")){
        if(any(!svd==svd.0.t)) yl<-"" else yl<-s
        plot(svd$inoculum, svd[,s],
             type="l", lwd=2, log="x",
             main="", axes=F,
             ylim=c(0,max(1,max(svd[,s]))),
             xlab="", ylab=yl)
        axis(2)
    }
    par(mar=c(1, 2, 1, 0) + 0.1)
    axis(1)
}
rm(svd,s,yl)
mtext("(A) default", adj=0.1, outer=T)
mtext("(B) EAko", adj=0.35, outer=T)
mtext("(C) EBko", adj=0.65, outer=T)
mtext("(D) ECko", adj=0.9, outer=T)
mtext("Inoculum size", adj=0.5, side=1, outer=T, line=3)
mtext("Trickle", adj=0.5, side=3, outer=T, line=3)
```

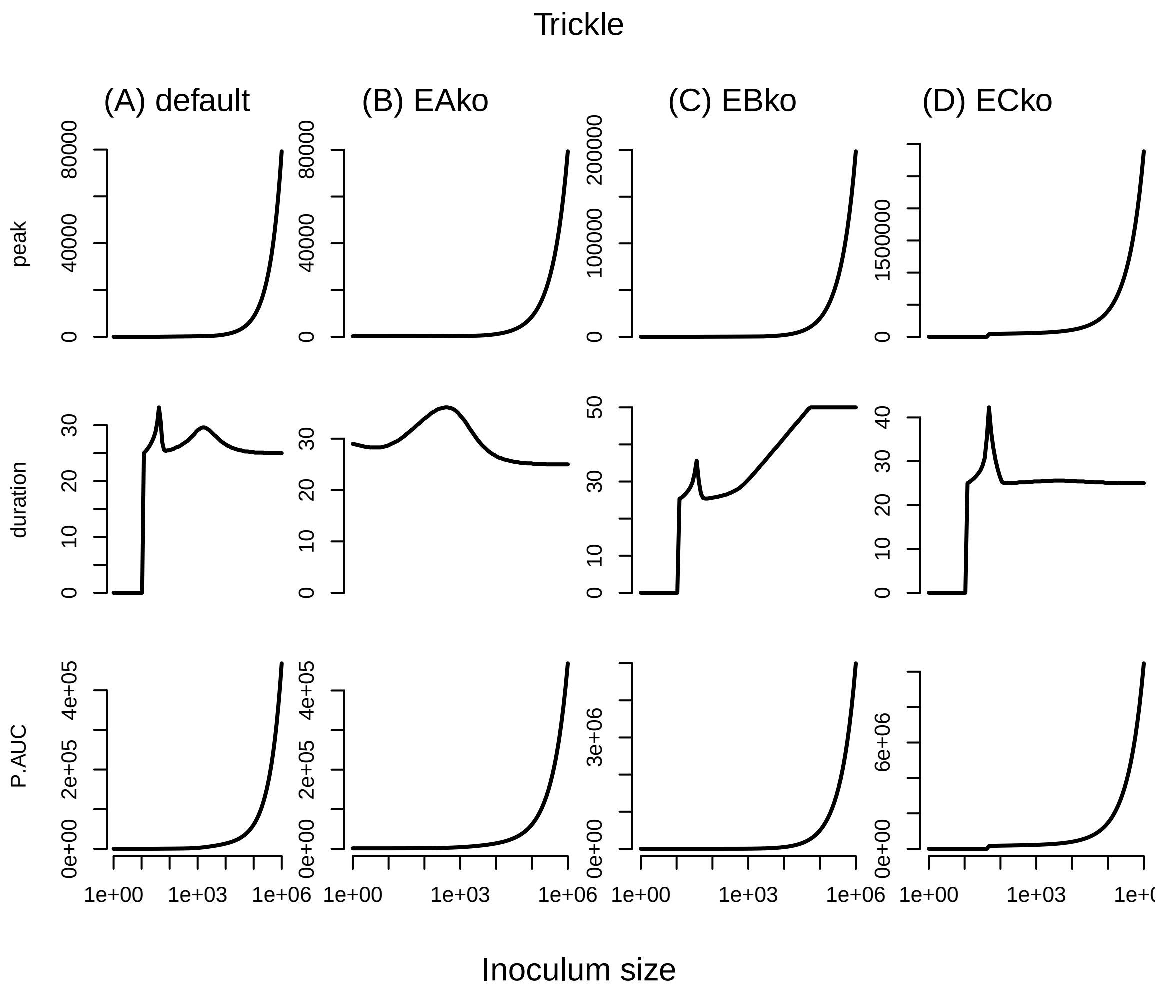

```
par(op);rm(op)
```

For continuous ("trickle") inoculations for non-replicating parasites ("worms") we obtain:

```
svd.0.w.t <-
    stats.vs.dose(inocula=inoc.seq,
                  schedule.type="trickle",
                  params.0=pars.0.w)
svd.EAko.w.t <-
    stats.vs.dose(inocula=inoc.seq,
                  schedule.type="trickle",
                  params.0=pars.EAko.w)
svd.EBko.w.t <-
    stats.vs.dose(inocula=inoc.seq,
                  schedule.type="trickle",
                  params.0=pars.EBko.w)
svd.ECko.w.t <-
    stats.vs.dose(inocula=inoc.seq,
                  schedule.type="trickle",
                  params.0=pars.ECko.w)
```

The following plots these statistics by knockout:

```
par(mfcol=c(3,4), omi=c(1,1,1,0), mar=c(1, 4, 1, 0) + 0.1, xpd=T)->op
for(svd in list(svd.0.w.t, svd.EAko.w.t, svd.EBko.w.t, svd.ECko.w.t)){
    for(s in c("peak", "duration", "P.AUC")){
        if(any(!svd==svd.0.w.t)) yl<-"" else yl<-s
        plot(svd$inoculum, svd[,s],
             type="l", lwd=2, log="x",
             main="", axes=F,
             ylim=c(0,max(1,max(svd[,s]))),
             xlab="", ylab=yl)
        axis(2)
    }
    par(mar=c(1, 2, 1, 0) + 0.1)
    axis(1)
}
rm(svd,s,yl)
mtext("(A) default", adj=0.1, outer=T)
mtext("(B) EAko", adj=0.35, outer=T)
mtext("(C) EBko", adj=0.65, outer=T)
mtext("(D) ECko", adj=0.9, outer=T)
mtext("Inoculum size", adj=0.5, side=1, outer=T, line=3)
mtext("Trickling Worms", adj=0.5, side=3, outer=T, line=3)
```

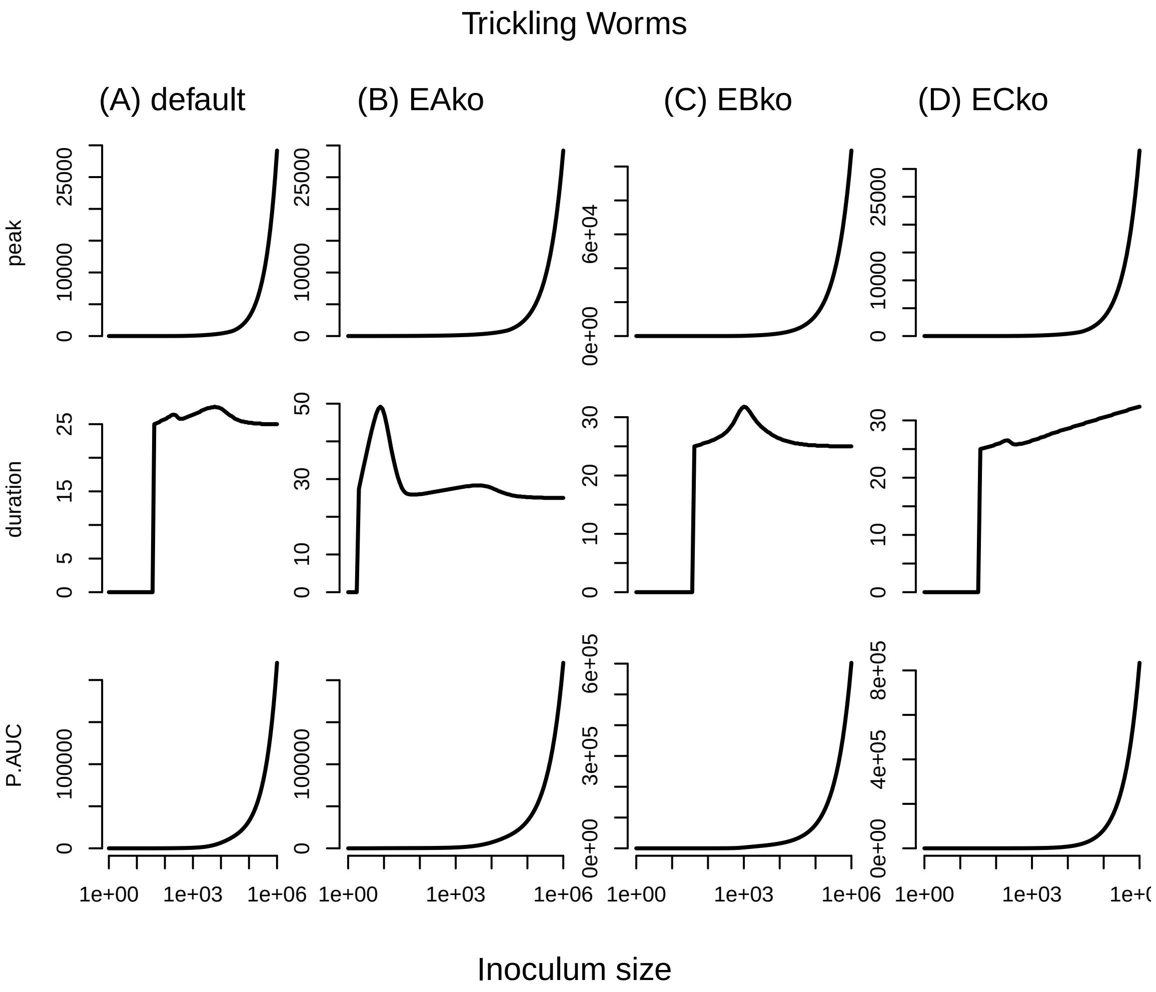


```
par(op);rm(op)
```

Infection summary statistics (duration, success, peak, and P.AUC) versus dose in stochastic simulations (Figure 4, 5, S4, and S5)

To estimate the probability of infection and distribution of infection durations predicted by the **stochastic** version of our model, we conduct multiple (`repl`) stochastic simulations, extract its duration, and check if the parasite load is larger than 0 at observation times `t.observe`. The following function implements this procedure:

```
stats.vs.dose.stoch <- function(repl=10,
                                model="dose.model", # or "alt.dose.model"
                                inocula=c(20,50,100,200,500,1000),
                                t.max=50, t.observe=c(10,20),
                                params.0=pars.0){

    if(model=="dose.model") dm <- dose.model
    if(model=="alt.dose.model") dm <- alt.dose.model

    output <- data.frame(sim.no=rep(1:repl, length(inocula)),
                         inoculum=rep(inocula, each=repl),
                         peak=NA,
```

```
                         P.AUC=NA,
                         duration=NA)

    for(t.o in t.observe) output <- cbind(output, NA)
    dimnames(output)[[2]][6:(5+length(t.observe))] <- paste0("success_", t.observe)
    attr(output, "parameters") <- params.0
    attr(output, "t.max") <- t.max
    attr(output, "t.observe") <- t.observe

    for(i in inocula){
        e <- list(max.dosing.rate=i/t.bolus,
                  duration=t.bolus,
                  schedule=c(0))
        for(r in 1:repl){
            sim.i <- dm(exposure=e,
                        tmax = t.max,
                        params = params.0,
                        deterministic=F)

            output[output$sim.no==r & output$inoculum==i, "peak"] <-
                extract.stats(sim=sim.i,
                              what="peak")
            output[output$sim.no==r & output$inoculum==i, "P.AUC"] <-
                extract.stats(sim=sim.i,
                              what="P.AUC")
            d <- extract.stats(sim=sim.i,
                               what="duration")
            output[output$sim.no==r & output$inoculum==i, "duration"] <- d

            for(t.o in t.observe){
                output[output$sim.no==r & output$inoculum==i,
                       paste0("success_",t.o)] <-
                    (sim.i[sim.i$time>=t.o, "P"][1] > 0)
            }
        }
    }
    output
}
```

The following command runs this for 200 replicates:

```
svd.stoch <- stats.vs.dose.stoch(repl=200,
                                 inocula=c(1,2,5,10,20,50,100,200,500,
                                           1000,2300,5000,10000),
                                 t.max=40,
                                 t.observe=seq(1, 20, by=1))
```

This plots the distribution of infection durations against the inoculum size as in Figure 4:

```
ggplot(data = svd.stoch, aes(x = inoculum, y = duration, group = inoculum)) +
    geom_violin() + geom_point(aes(), alpha=.1) +
    scale_x_log10("Inoculum size") +
    scale_y_continuous("Infection duration")
```

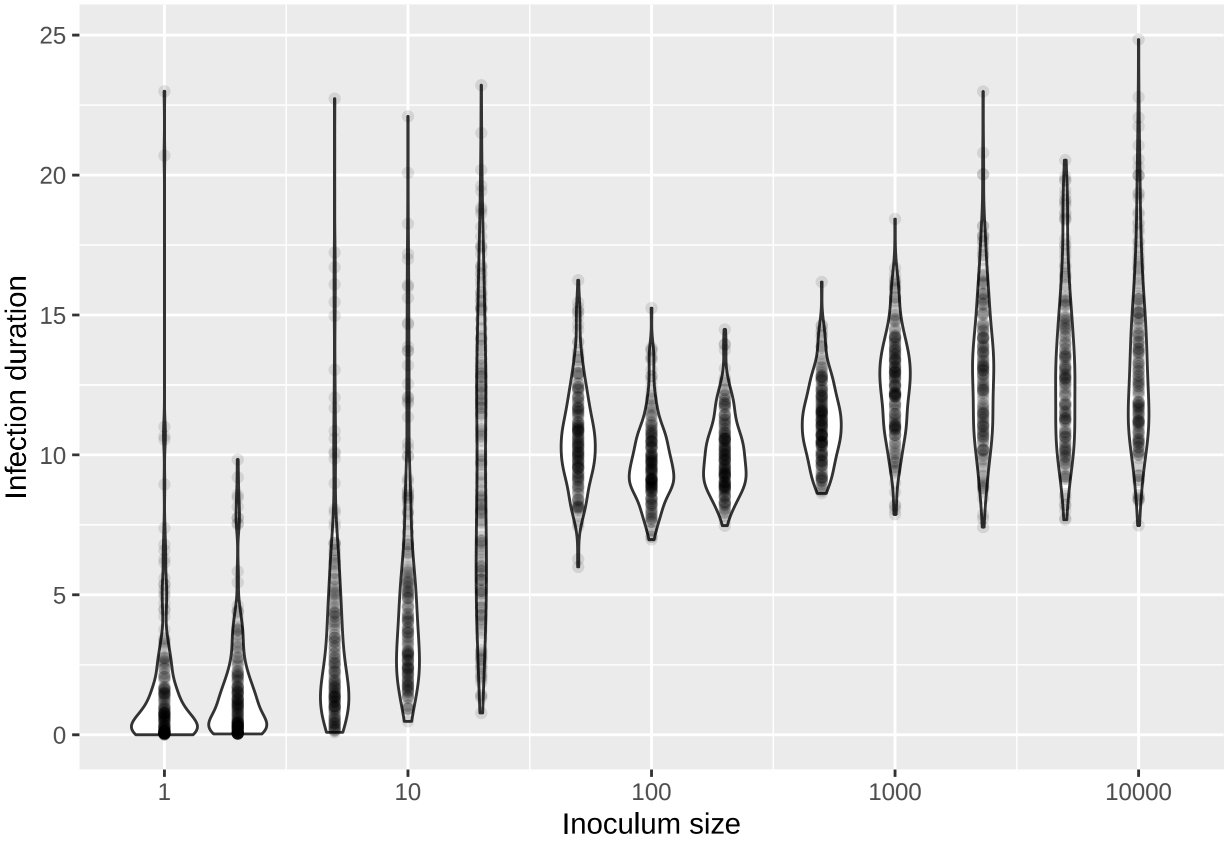

This plots infection success against the inoculum size and time of observation as in Figure 5:

```
t.obs <- attr(svd.stoch, "t.observe")
success.matrix <- matrix(0,
                         ncol=length(t.obs),
                         nrow=length(unique((svd.stoch$inoculum))))
for(i in 1:length(t.obs)){
    success.matrix[,i] <-
        c(by(as.numeric(svd.stoch[,paste0("success_", t.obs[i])]),
             svd.stoch$inoculum, mean))
}
filled.contour(x=log10(unique(svd.stoch$inoculum)),
               y=t.obs,
               z=success.matrix,
               xlab="Inoculum size",
               ylab="Time of observation (days)",
               levels=seq(0,1,length=11),
               col=gray(0:10/10),
               key.title = title(main = "Infection\nsuccess"),
               plot.axes={axis(1, 1:3, expression(10,10^2,10^3))
                   axis(2, t.obs)},
               frame.plot=F)
```

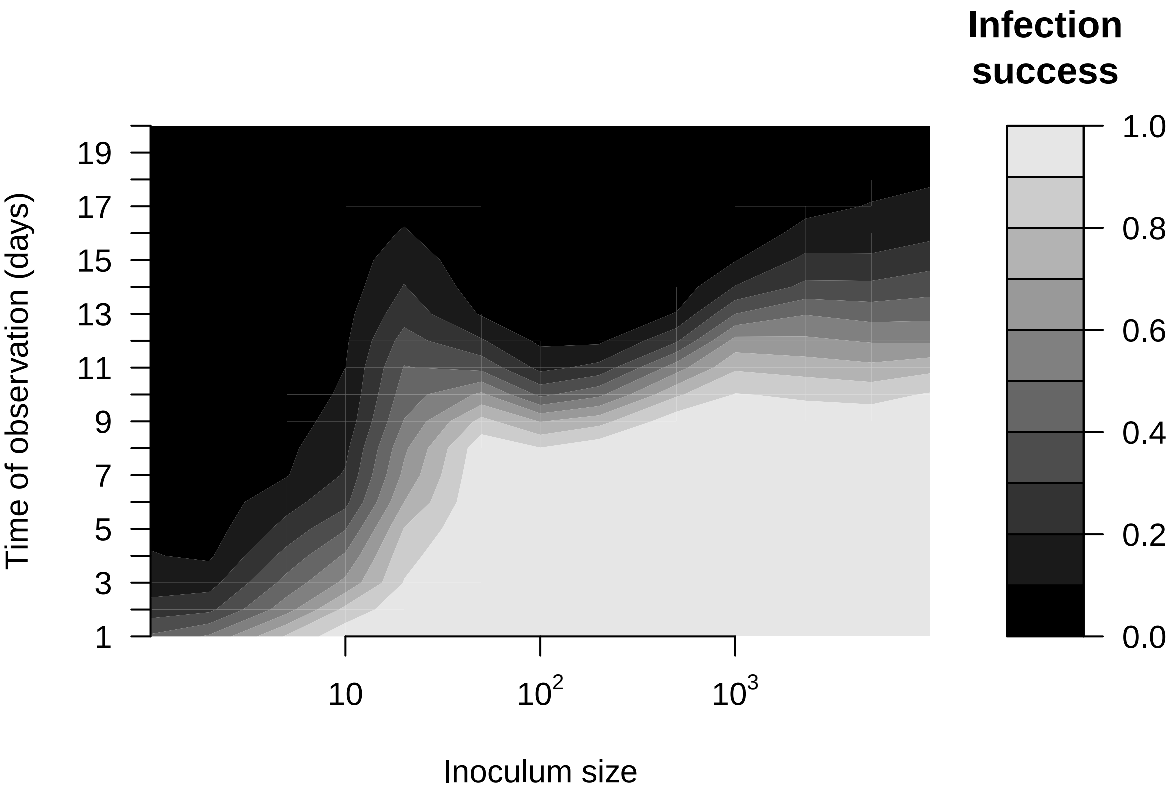

This plots the distribution of peak parasite load against the inoculum size as in Figure S4:

```
ggplot(data = svd.stoch, aes(x = inoculum, y = peak, group = inoculum)) +
    geom_violin() + geom_point(aes(), alpha=.1) +
    scale_x_log10("Inoculum size") +
    scale_y_continuous("Peak parasite load")
```

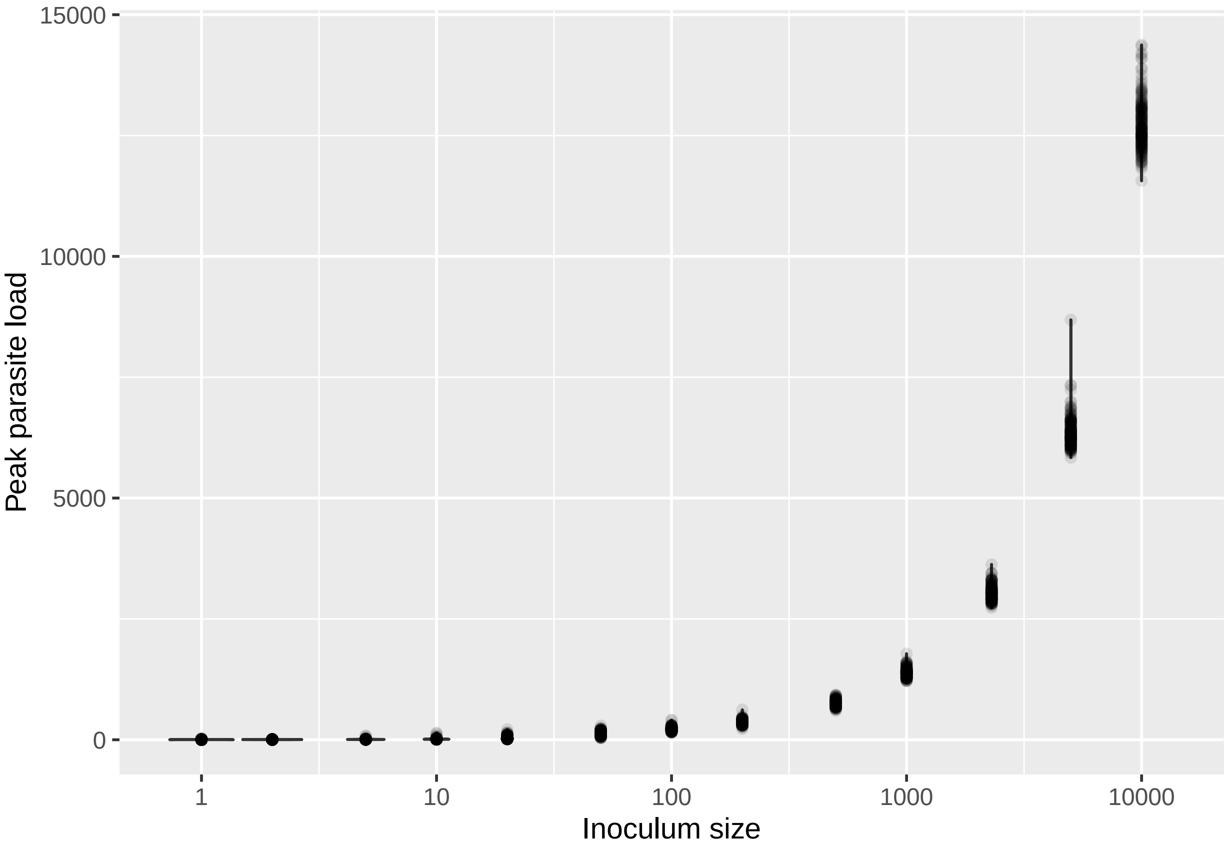

This plots the distribution of area under the parasite load curve `P.AUC` against the incoulum size as in Figure S5:

```
ggplot(data = svd.stoch, aes(x = inoculum, y = P.AUC, group = inoculum)) +
    geom_violin() + geom_point(aes(), alpha=.1) +
    scale_x_log10("Inoculum size") +
    scale_y_continuous("P.AUC")
```

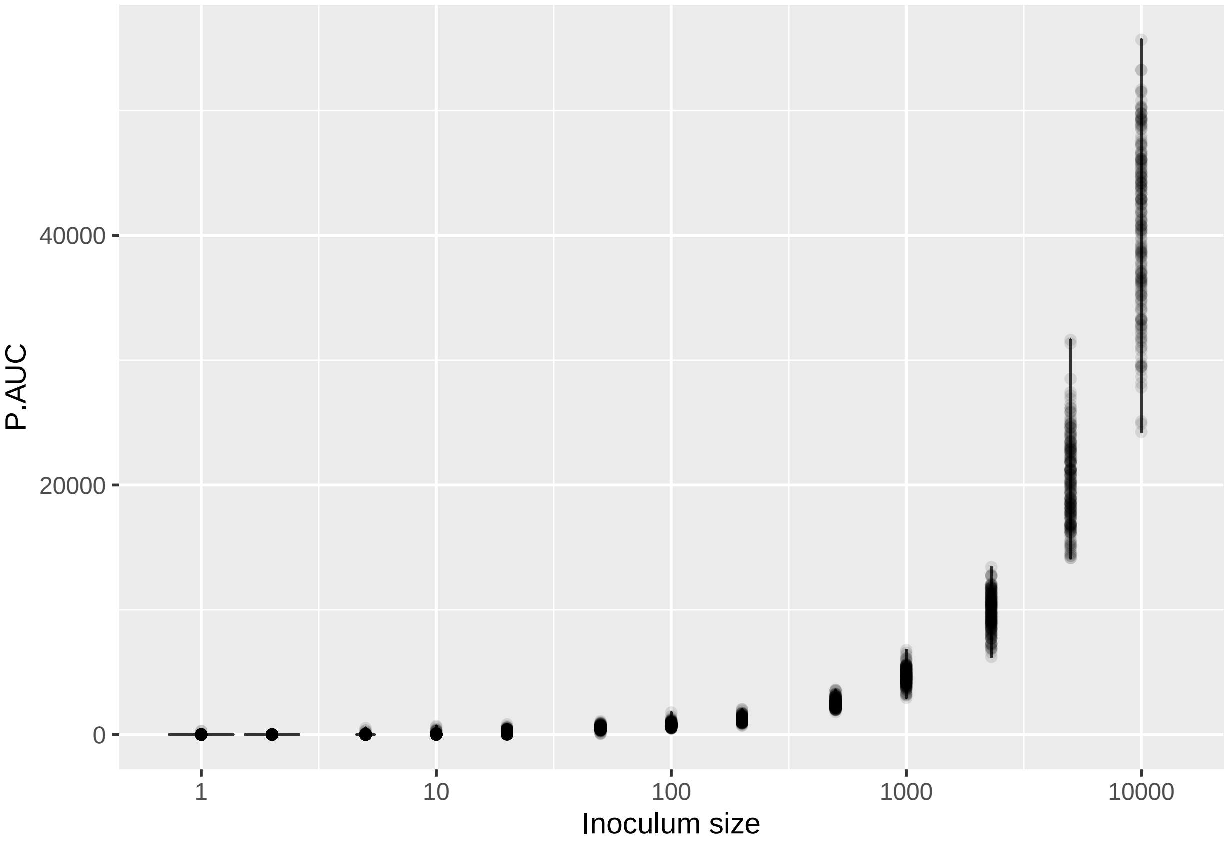

Variation in infection outcome (Figure S3)

The stochastic version of our model can displays large variations in the infection dynamics similar to that shown experimentally in Duneau et al 2017 eLife. To illustrate this variable, at times even dichotomous, dynamics, we ran multiple (`repl`) **stochastic** simulations and plot the time courses for multiple doses.

We expect the largest variations in infection dynamics for the parameter set `pars.EBko`, in which the second tier responses are knocked out. This is due to the fact that for this parameterization there is only a single peak in the relationship between the infection duration and the inoculum that is higher and sharper than for the default parameters.

The inoculum, for which the variation in infection dynamics is maximal, can be read off Figure 2 (above) that shows the duration distribution versus inoculum dose: 23. Consequently, we define values around that number as default inocula in these simulations:

```
sims.duneau <- function(repl=10,
                        t.max=50,
                        inocula=c(5,23,100),
                        params=pars.EBko){

    sims <- NULL
    for(i in inocula){
        for(r in 1:repl){
            s <- dose.model(exposure=list(max.dosing.rate=i/t.bolus,
                                          duration=t.bolus,
                                          schedule=c(0)),
                            tmax=t.max,
                            params=params,
```

```
                              deterministic=F)
            sims <- rbind(sims,
                          cbind(r=r, inoculum=i,s))
        }
    }
    sims
}
```

The following commands produce the time courses by inoculum dose:

```
sims.duneau.out <- sims.duneau(r=50)

require(ggplot2)
fig.duneau <-
    ggplot(data=sims.duneau.out,
           aes(x=time,y=P,group=r))+
    facet_grid(.~inoculum,scales="free_y")+
    geom_line(color="black", alpha=1/10) +
    scale_x_continuous(name="Time since inoculation (days)",
                       limits=c(.1,max(sims.duneau.out$time))) +
    scale_y_log10("Parasite burden")
print(fig.duneau)
```

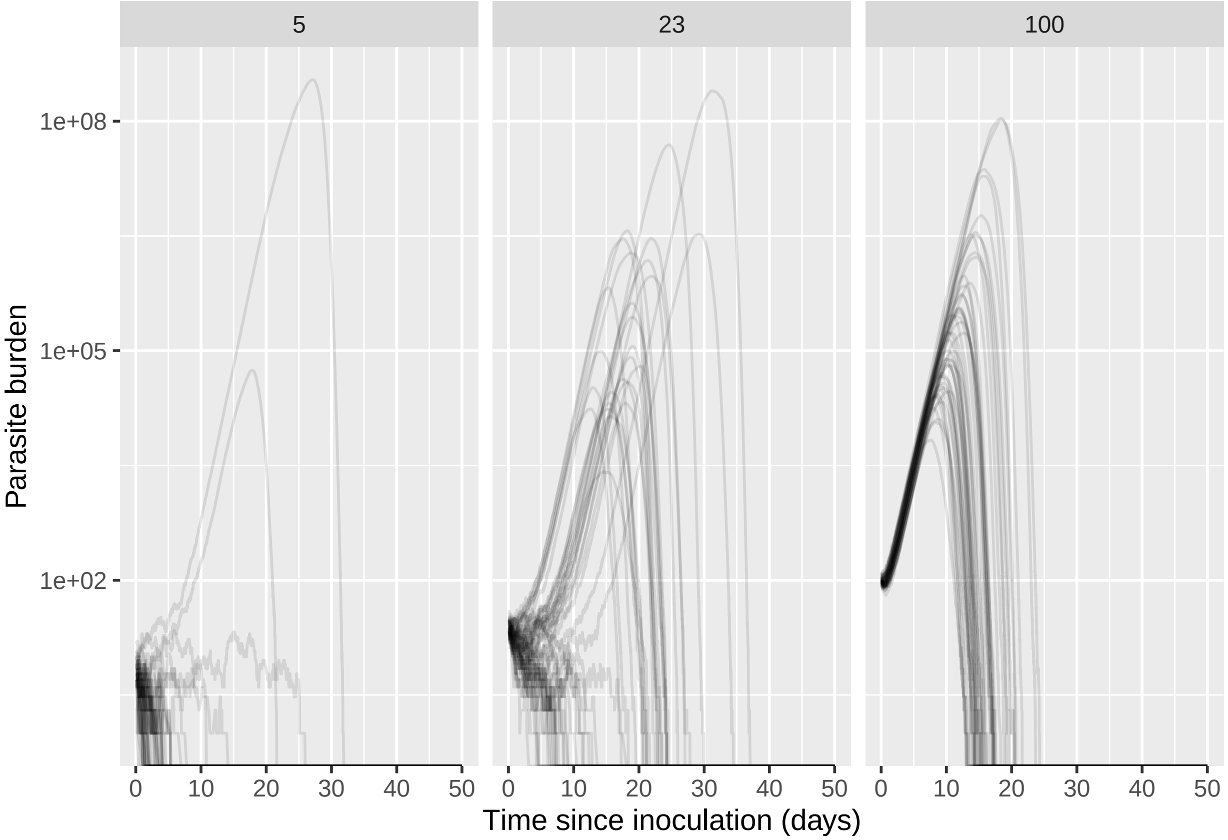

At an inoculum dose of 23 the infection outcome is bimodal, leading to either fast parasite clearance or a burst in parasite load and longer infection duration.

Model with alternative formulation of the three immune response tiers

To assess the robustness of our findings to the specific formulation of the model we developed an alternative model that features many alternative population dynamical implementations of the interaction between the parasite and the three immune response tiers. In particular, the alternative model assumes:

1) Two compartments for the parasite, one "outside" the host, `P.outside`, that is seeded by the inoculum, and one inside the host, `P`. The barrier defense is now assumed to clear the parasite population in the outside compartment. Furthermore, the parasite is assumed not to replicate in the outside compartment.
2) The innate defense is now modelled similar to Antia et al 1994 JTB and Handel et al 2017 PLOS Comp Biol, consisting of a finite pool of effectors ("macrophages") that need to be activated by the parasite to become effective (`sigmaB*(KEB - EB)*P/(hB+P)` instead of `sigmaB*(1 - EB/KEB)*EB*P/(hB+P)`).
3) We also assume that the adaptive immune response is not only triggered by the parasite but further requires a sizable innate reponse to be elicited, similar to the model by Handel et al PLOS Comp Biol 2018 (`sigmaC*(1 - EC/KEC)*EC*P*EB/(hC+P)` instead of `sigmaC*(1 - EC/KEC)*EC*P/(hC+P)`).

Overall, our results are robust to this large set of changes.

The following function implements this alternative model:

```
alt.dose.model <- function(exposure=exp.0,
                           tmax=30,
                           params=alt.pars.0,
                           deterministic=T){

    ## exposure rate function
    eta.1 <- function(t, e){
        with(as.list(e),{
            if(any(schedule <= t & t < schedule+duration))
                out <- max.dosing.rate
            else out <- 0
            return(out)
        })
    }
    eta <- function(t.vec, e=exposure) {
        sapply(t.vec, function(t) eta.1(t, e=e))
    }

    if(deterministic){
        ## define derivatives for ODEs:
        derivs <- function(t,x,p){
            P.outside <- x[1]
            P <- x[2]
            EA <- x[3]
            EB <- x[4]
            EC <- x[5]
            with(as.list(p),{
                dP.outside <- eta(t) - P.outside*gammaA*EA - m*P.outside
                ## P.outside ("outside parasites") do not replicate and
                ## are affected only by barrier defense EA
                dP <- m*P.outside + P*r  - P*(gammaB*EB + gammaC*EC)
                dEA <- sigmaA*(1-EA/EA0) - gammaA*P.outside*EA
                ##dEB <- sigmaB*(1 - EB/KEB)*EB*P/(hB+P)
```

```
            dEB <- sigmaB*(KEB - EB)*P/(hB+P)
            ##dEC <- sigmaC*(1 - EC/KEC)*EC*P/(hC+P)
            dEC <- sigmaC*(1 - EC/KEC)*EC*P*EB/(hC+P) # EC needs EB

            list(c(dP.outside, dP, dEA, dEB, dEC))
        })
    }

    ## solve ODEs:
    require(deSolve)
    P.outside.init <- 0; P.init <- 0
    inits <- c(P.outside=P.outside.init, P=P.init, EA=params[["EA0"]],
               EB=params[["EB0"]], EC=params[["EC0"]])
    times <- seq(0, tmax, length=501)
    output <-as.data.frame(lsoda(inits, times, derivs, params))
}

if(!deterministic){
    require(adaptivetau)
    P.outside.init <- 0; P.init <- 0
    inits <- c(P.outside=P.outside.init, P=P.init, EA=params[["EA0"]],
               EB=params[["EB0"]], EC=params[["EC0"]])

    transitions <-
        ssa.maketrans(c("P.outside", "P", "EA", "EB", "EC"),
                      rbind("P.outside", +1), # inoculation of P.outside
                      rbind("P.outside", -1, "P", +1), # migration from
                                                       # P.outside to P
                      rbind("P.outside", -1, "EA", -1), # deathP and EA
                                                        # by killing interaction
                      rbind("P", +1), # growthP
                      rbind("P", -1), # deathP by killing interaction
                      rbind("P", -1), # deathP by killing interaction
                      rbind("EA", +1), # replenishmentEA
                      rbind("EA", -1), # stop-replenishmentEA
                                       # (needed to avoid negative rates)
                      rbind("EB", +1), # stimulationEB
                      rbind("EB", -1), # unstimulationEB
                                       # (needed to avoid negative rates)
                      rbind("EC", +1), # growthEC
                      rbind("EC", -1)) # deathEC

    trans.rates <- function(x, p, t){
        P.outside <- x[1]
        P <- x[2]
        EA <- x[3]
        EB <- x[4]
        EC <- x[5]
        with(as.list(p),{
            rates <-
                c(eta(t),
                  P.outside * m,
                  gammaA*P.outside*EA,
```

```
                      P * r,
                      gammaB*P*EB,
                      gammaC*P*EC,
                      sigmaA,
                      sigmaA*EA/EA0,
                      sigmaB*KEB*P/(hB+P),
                      sigmaB*EB*P/(hB+P),
                      sigmaC*EC*P*EB/(hC+P),
                      sigmaC*EC*P*EB/(hC+P)*EC/KEC)
                names(rates) <- NULL
                return(rates)
            })
        }

        output <-
            as.data.frame(ssa.adaptivetau(inits, transitions,
                                          trans.rates, params,
                                          tf=tmax))
    }

    ## print output:
    output
}
```

The alternative default parameters, `alt.pars.0`, are identical to the original default parameters, `pars.0`, except for the new additional parameter `m` that denotes the rate at which parasites transit from the outside compartment, `P.outside`, to inside, `P`, and a 10'000-fold reduction of `sigmaC` that was necessary to keep the stimulation of the adaptive responses, `EC`, similarly fast as in the original model (because `EB` now appears as a factor in that term and is maximally `KEB`=10'000):

```
alt.pars.0 <- c(m=1, r=1, gammaA=0.015, gammaB=0.00012, gammaC=0.05,
                sigmaA=30, sigmaB=40, sigmaC=5e-5, hB=1000, hC=1000, KEB=1e4, KEC=1e6,
                EA0=100, EB0=1, EC0=1)
```

Infection summary statistics across inocula in alternative model (Figure S6)

For bolus inoculations with default parameters and parameters describing immune knockouts, in the alternative model we obtain:

```
alt.svd.0 <-
    stats.vs.dose(model="alt.dose.model",
                  inocula=inoc.seq,
                  params.0=alt.pars.0)

alt.pars.EAko <- alt.pars.0
alt.pars.EAko[c("gammaA","EA0")] <- c(0.0,1e-3)
alt.svd.EAko <-
    stats.vs.dose(model="alt.dose.model",
                  inocula=inoc.seq,
                  params.0=alt.pars.EAko)

alt.pars.EBko <- alt.pars.0
alt.pars.EBko[c("gammaB","EB0")] <- c(0.0,0.0)
alt.svd.EBko <-
    stats.vs.dose(model="alt.dose.model",
```

```
                  inocula=inoc.seq,
                  params.0=alt.pars.EBko)

alt.pars.ECko <- alt.pars.0
alt.pars.ECko[c("gammaC","EC0")] <- c(0.0,0.0)
alt.svd.ECko <-
    stats.vs.dose(model="alt.dose.model",
                  inocula=inoc.seq,
                  params.0=alt.pars.ECko)
```

The following plots these statistics by knockout:

```
par(mfcol=c(3,4), omi=c(1,1,1,0), mar=c(1, 4, 1, 0) + 0.1, xpd=T)->op
for(svd in list(alt.svd.0, alt.svd.EAko, alt.svd.EBko, alt.svd.ECko)){
    for(s in c("peak", "duration","P.AUC")){
        if(any(!svd==alt.svd.0)) yl<-"" else yl<-s
        plot(svd$inoculum, svd[,s],
             type="l", lwd=2, log="x",
             main="", axes=F,
             ylim=c(0,max(1,max(svd[,s]))),
             xlab="", ylab=yl)
        axis(2)
    }
    par(mar=c(1, 2, 1, 0) + 0.1)
    axis(1)
}
rm(svd,s,yl)
mtext("(A) default", adj=0.1, outer=T)
mtext("(B) EAko", adj=0.35, outer=T)
mtext("(C) EBko", adj=0.65, outer=T)
mtext("(D) ECko", adj=0.9, outer=T)
mtext("Inoculum size", adj=0.5, side=1, outer=T, line=3)
mtext("Bolus (alternative model)", adj=0.5, side=3, outer=T, line=3)
```

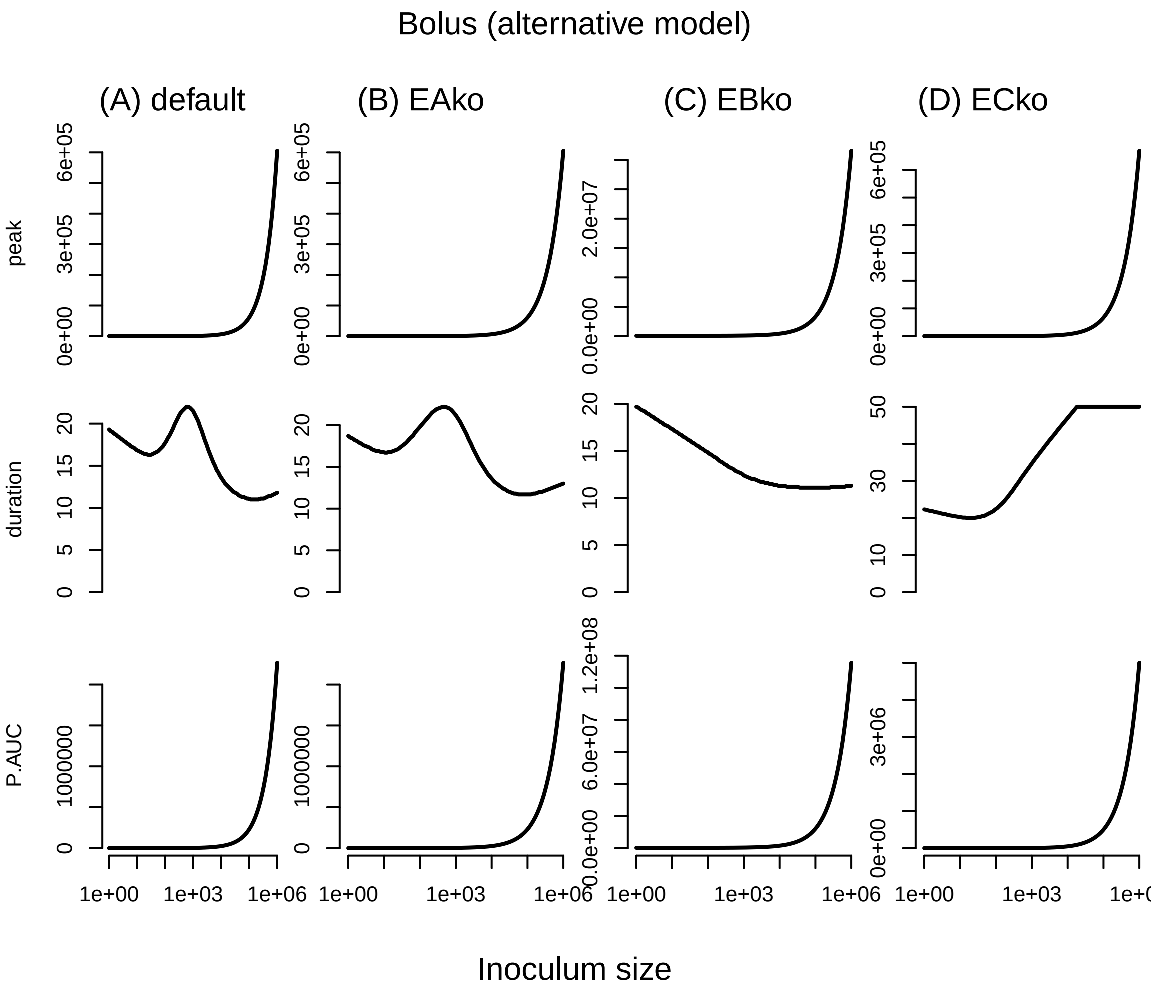


```
par(op);rm(op)
```

Infection duration and success versus dose in stochastic simulations in alternative model (Figure S7)

The following command runs this for 200 replicates:

```
alt.svd.stoch <-
    stats.vs.dose.stoch(repl=200,
                        model="alt.dose.model",
                        inocula=c(1,2,5,10,20,50,100,200,500,1000,2300,5000,10000),
                        t.max=40,
                        t.observe=seq(1, 20, by=1),
                        params.0=alt.pars.0)
```

This plots infection success against the inoculum size and time of observation for the alternative model (compare this to Figure 5):

```
t.obs <- attr(alt.svd.stoch, "t.observe")
alt.success.matrix <-
    matrix(0,
           ncol=length(t.obs),
           nrow=length(unique((alt.svd.stoch$inoculum))))
for(i in 1:length(t.obs)){
```

```
    alt.success.matrix[,i] <-
        c(by(as.numeric(alt.svd.stoch[,paste0("success_", t.obs[i])]),
             alt.svd.stoch$inoculum, mean))
}
filled.contour(x=log10(unique(alt.svd.stoch$inoculum)),
               y=t.obs,
               z=alt.success.matrix,
               xlab="Inoculum size",
               ylab="Time of observation (days)",
               levels=seq(0,1,length=11),
               col=gray(0:10/10),
               key.title = title(main = "Infection\nsuccess"),
               plot.axes={axis(1, 1:3, expression(10,10^2,10^3))
                   axis(2, t.obs)},
               frame.plot=F)
```

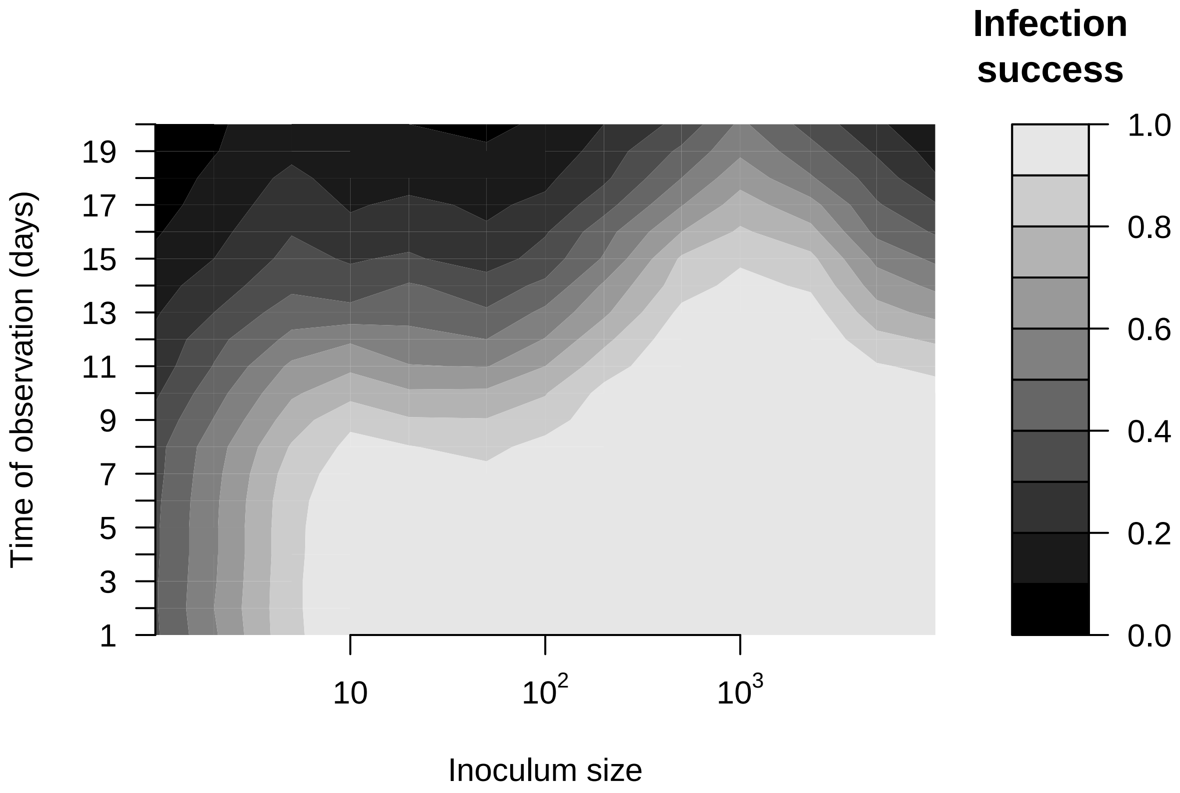

Supplement: S1 Code — (PDF) [file pcbi.1012652.s008.pdf]
